# Supplementary figures and images for: Sustainable fishing harvest rates for fluctuating fish and invertebrate stocks
Source: PLoS One. 2024 Sep 26;19(9):e0307836. doi: 10.1371/journal.pone.0307836 (PMC11426504; doi:10.1371/journal.pone.0307836)

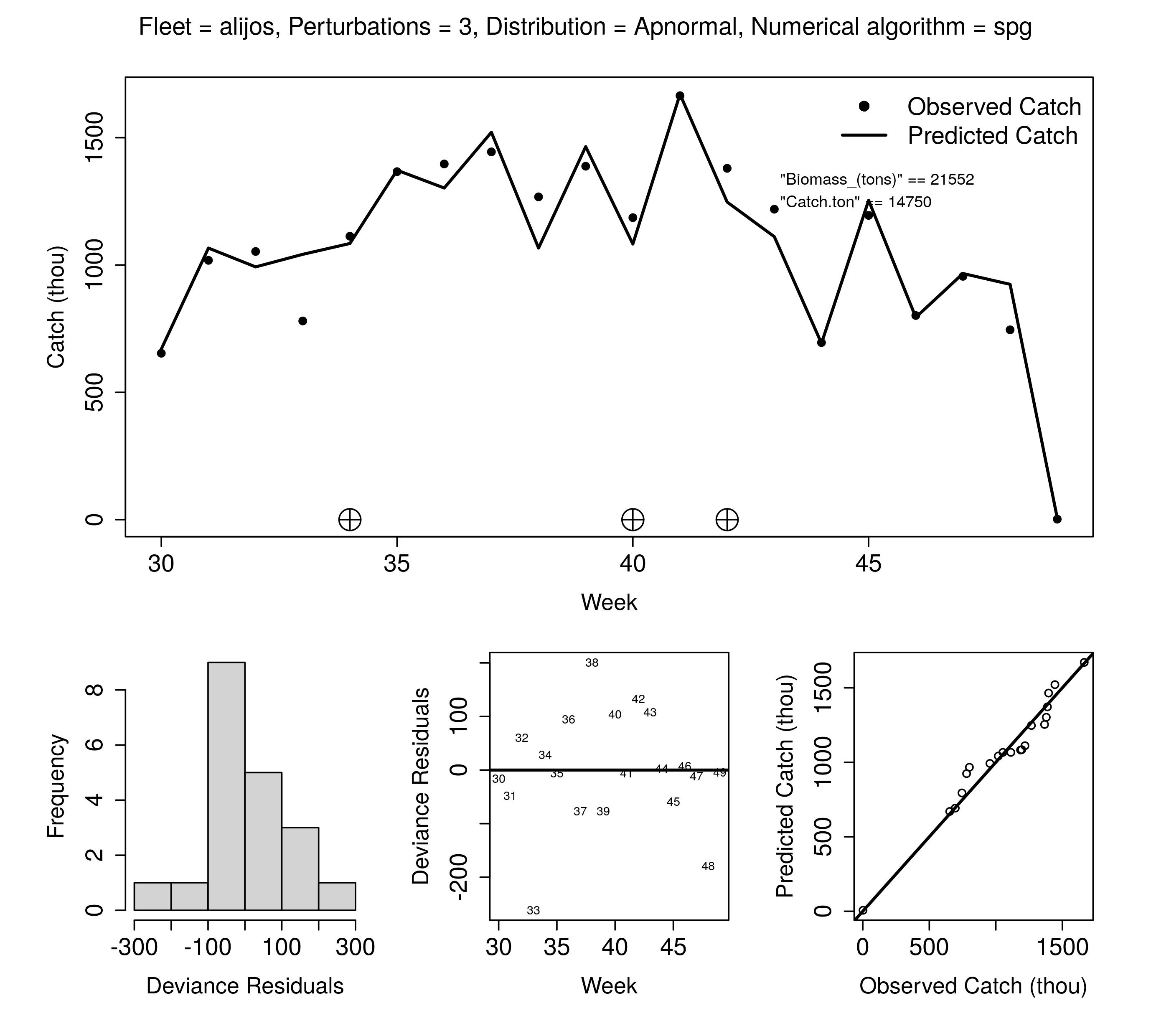

Supplement: S1 File — Model fit to data (top panel; dots: data; line: model) and residual diagnostics (three bottom panels; left: residual histogram; centre: residual cloud; right: quantile-quantile plot) for 22 fishing seasons of O. maya in Yucatan, Mexico. (ZIP) [file pone.0307836.s001.zip › FigS15CatDynMaya2014.jpg]

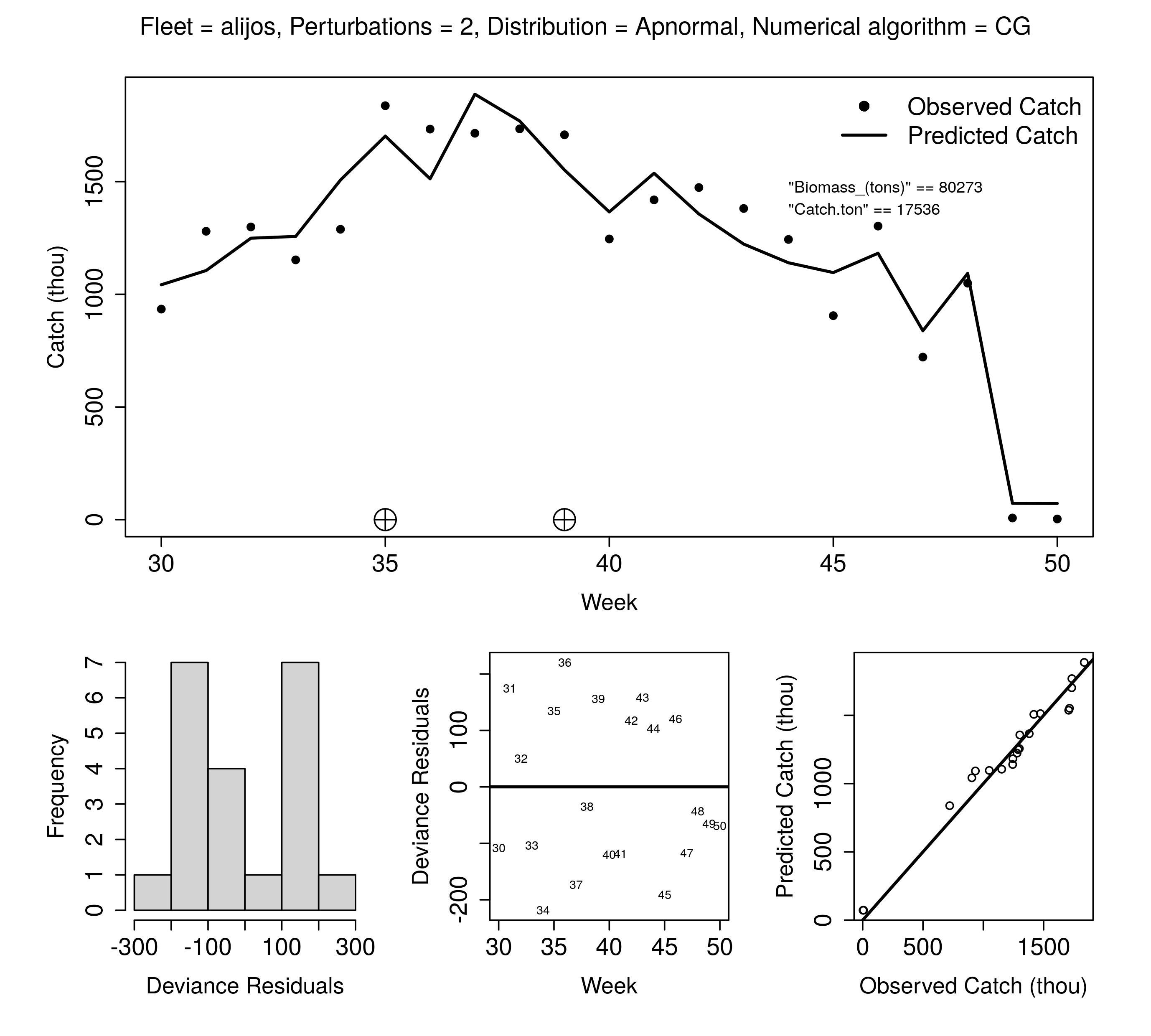

Supplement: S1 File — Model fit to data (top panel; dots: data; line: model) and residual diagnostics (three bottom panels; left: residual histogram; centre: residual cloud; right: quantile-quantile plot) for 22 fishing seasons of O. maya in Yucatan, Mexico. (ZIP) [file pone.0307836.s001.zip › FigS16CatDynMaya2015.jpg]

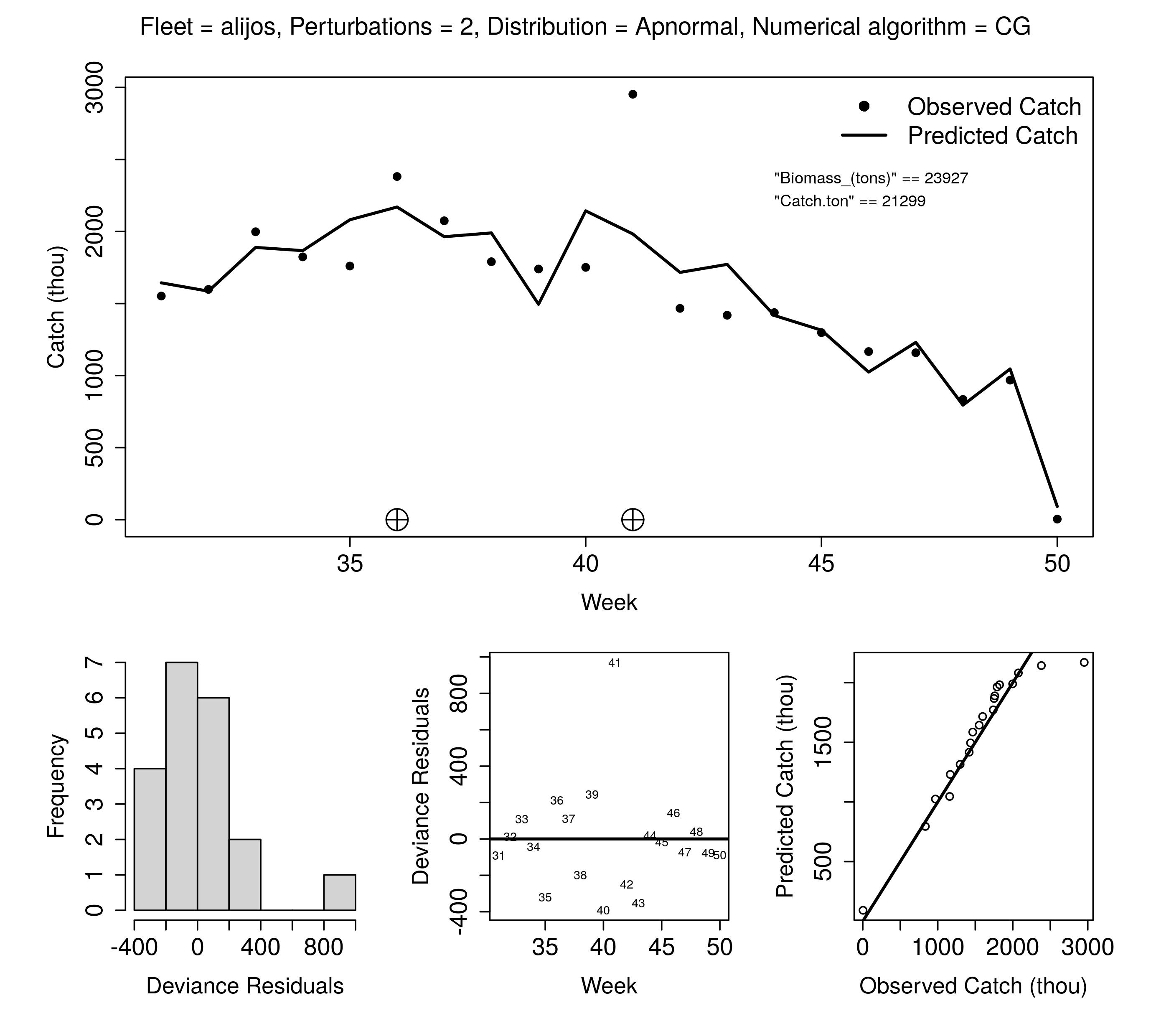

Supplement: S1 File — Model fit to data (top panel; dots: data; line: model) and residual diagnostics (three bottom panels; left: residual histogram; centre: residual cloud; right: quantile-quantile plot) for 22 fishing seasons of O. maya in Yucatan, Mexico. (ZIP) [file pone.0307836.s001.zip › FigS17CatDynMaya2016.jpg]

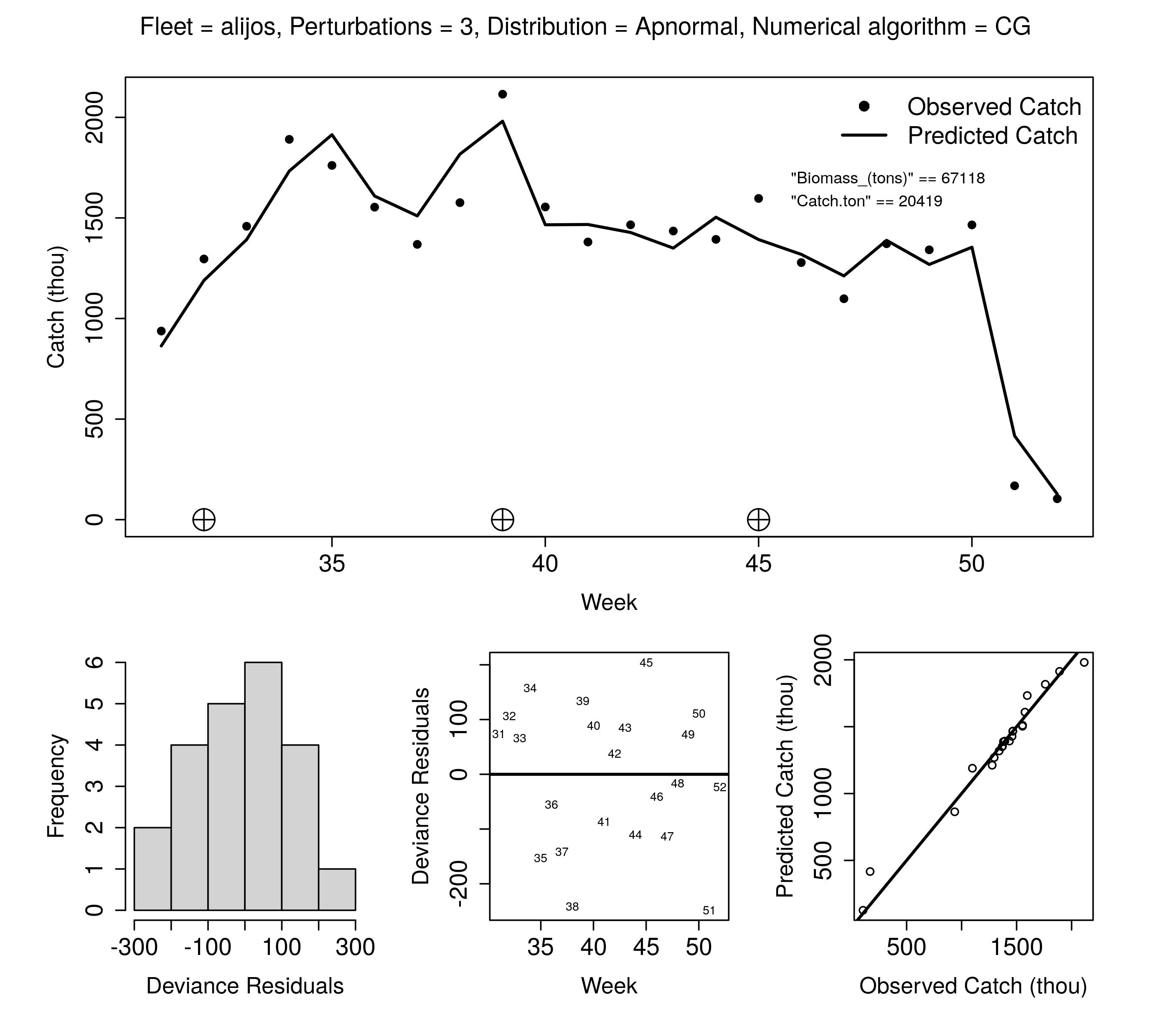

Supplement: S1 File — Model fit to data (top panel; dots: data; line: model) and residual diagnostics (three bottom panels; left: residual histogram; centre: residual cloud; right: quantile-quantile plot) for 22 fishing seasons of O. maya in Yucatan, Mexico. (ZIP) [file pone.0307836.s001.zip › FigS18CatDynMaya2017.jpg]

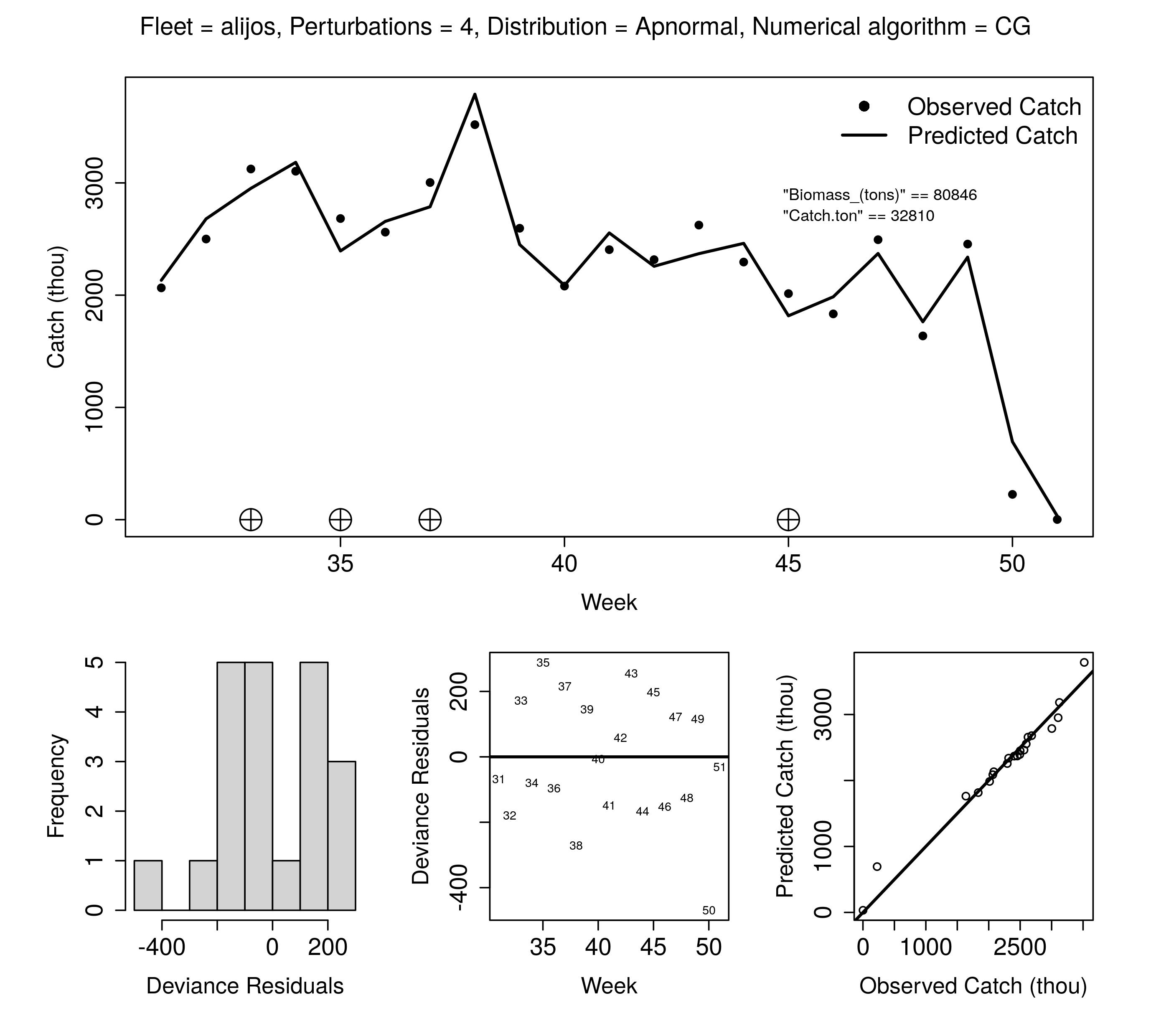

Supplement: S1 File — Model fit to data (top panel; dots: data; line: model) and residual diagnostics (three bottom panels; left: residual histogram; centre: residual cloud; right: quantile-quantile plot) for 22 fishing seasons of O. maya in Yucatan, Mexico. (ZIP) [file pone.0307836.s001.zip › FigS19CatDynMaya2018.jpg]

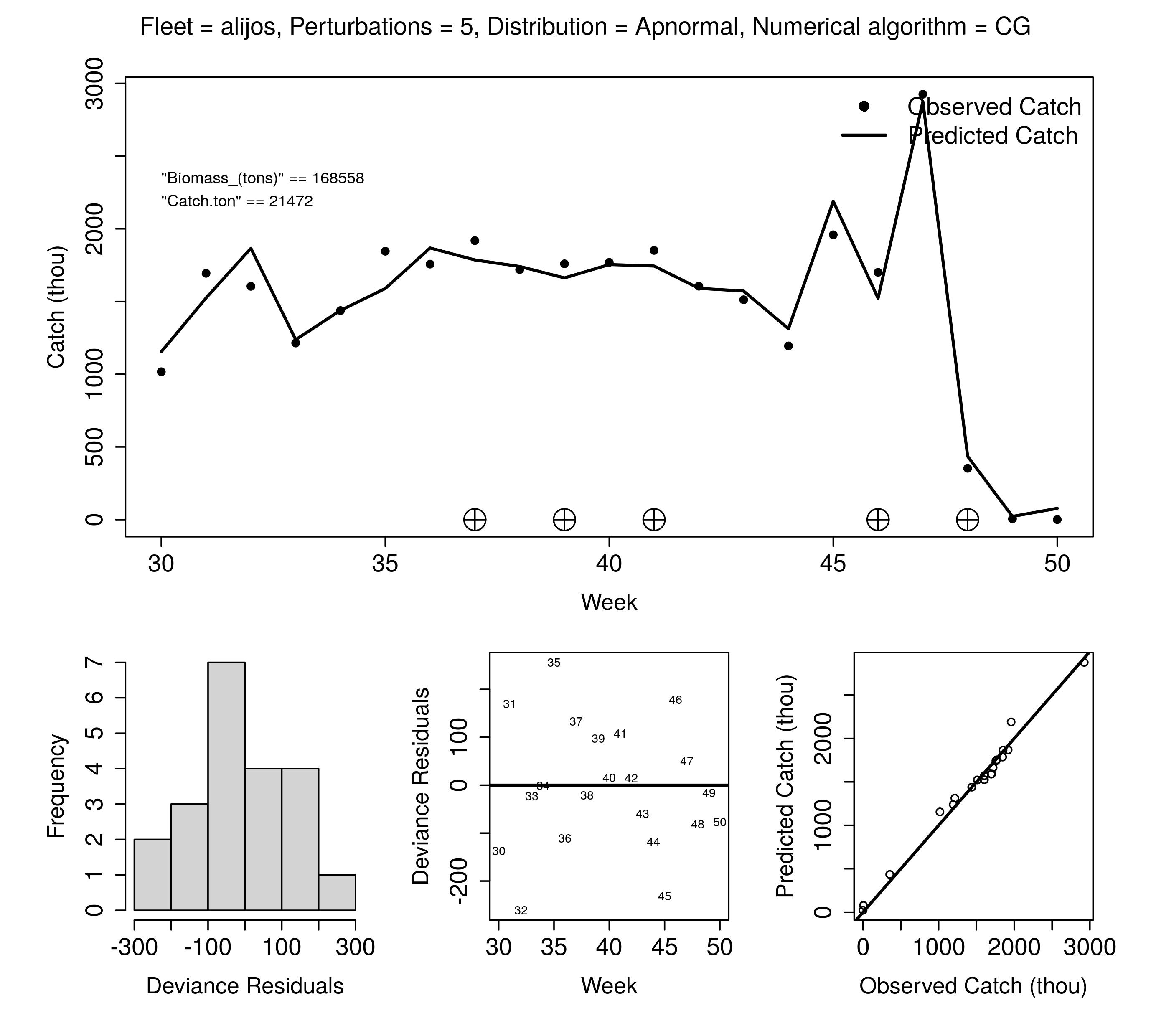

Supplement: S1 File — Model fit to data (top panel; dots: data; line: model) and residual diagnostics (three bottom panels; left: residual histogram; centre: residual cloud; right: quantile-quantile plot) for 22 fishing seasons of O. maya in Yucatan, Mexico. (ZIP) [file pone.0307836.s001.zip › FigS20CatDynMaya2019.jpg]

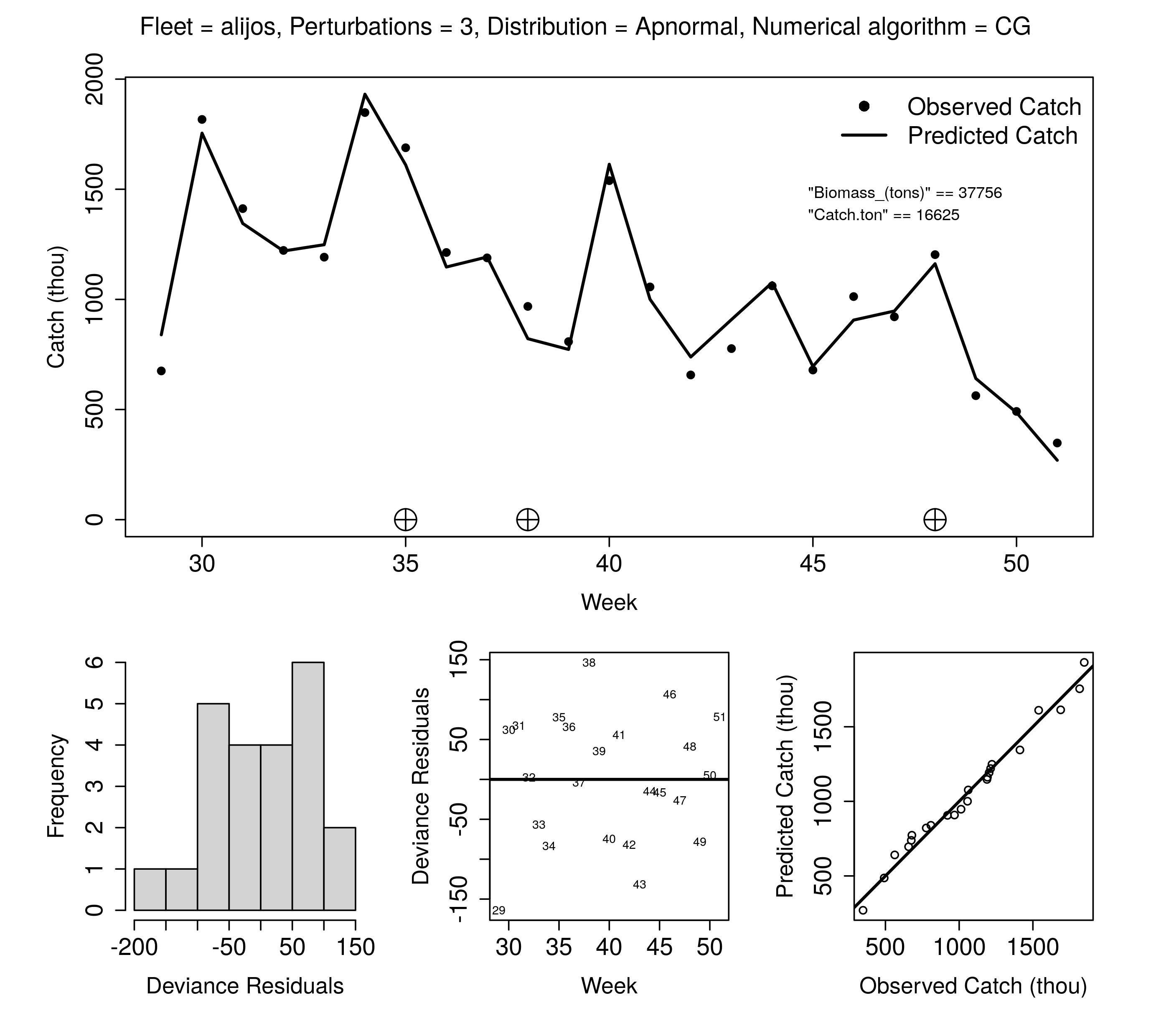

Supplement: S1 File — Model fit to data (top panel; dots: data; line: model) and residual diagnostics (three bottom panels; left: residual histogram; centre: residual cloud; right: quantile-quantile plot) for 22 fishing seasons of O. maya in Yucatan, Mexico. (ZIP) [file pone.0307836.s001.zip › FigS21CatDynMaya2020.jpg]

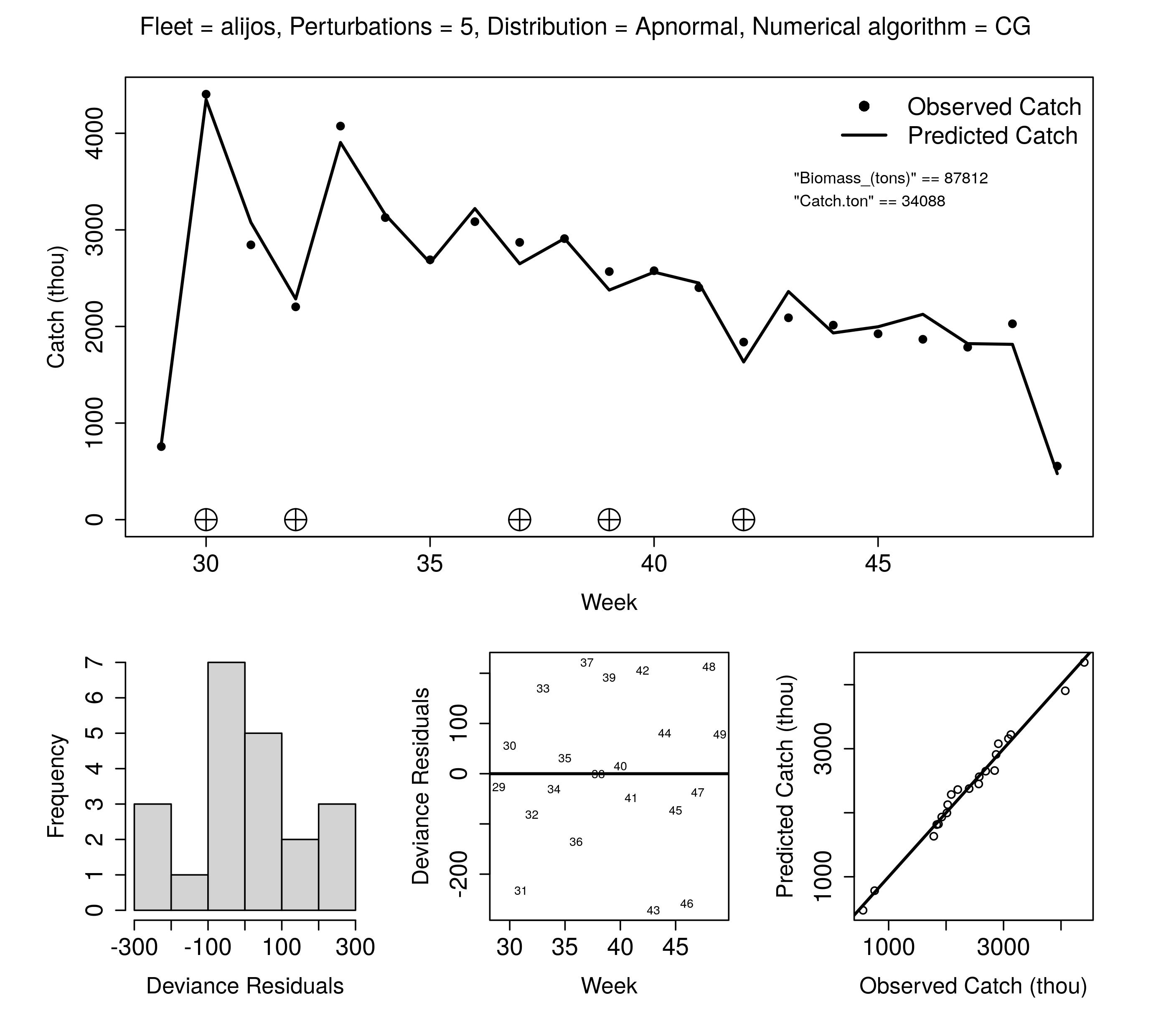

Supplement: S1 File — Model fit to data (top panel; dots: data; line: model) and residual diagnostics (three bottom panels; left: residual histogram; centre: residual cloud; right: quantile-quantile plot) for 22 fishing seasons of O. maya in Yucatan, Mexico. (ZIP) [file pone.0307836.s001.zip › FigS22CatDynMaya2021.jpg]

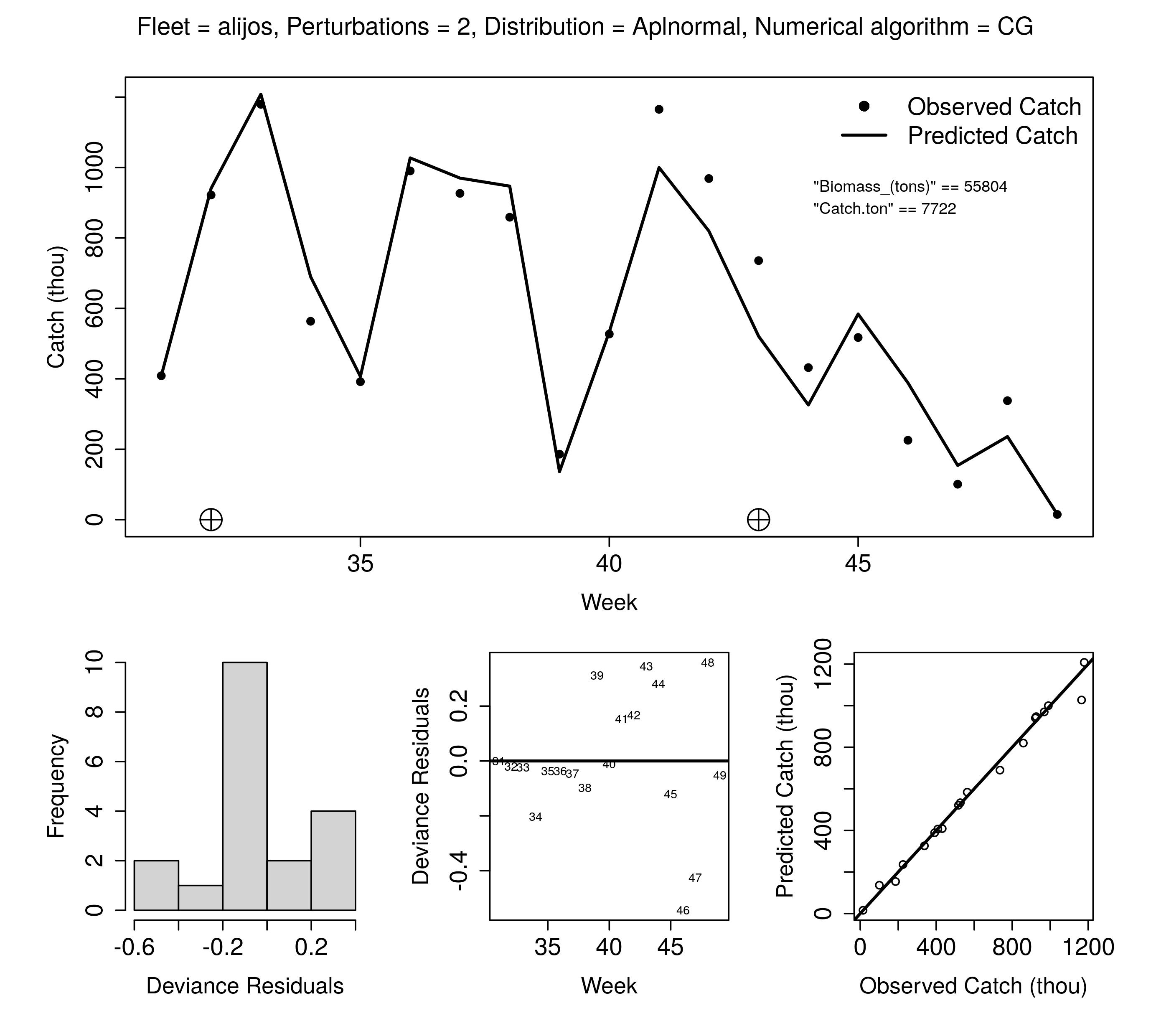

Supplement: S1 File — Model fit to data (top panel; dots: data; line: model) and residual diagnostics (three bottom panels; left: residual histogram; centre: residual cloud; right: quantile-quantile plot) for 22 fishing seasons of O. maya in Yucatan, Mexico. (ZIP) [file pone.0307836.s001.zip › FigS01CatDynMaya2000.jpg]

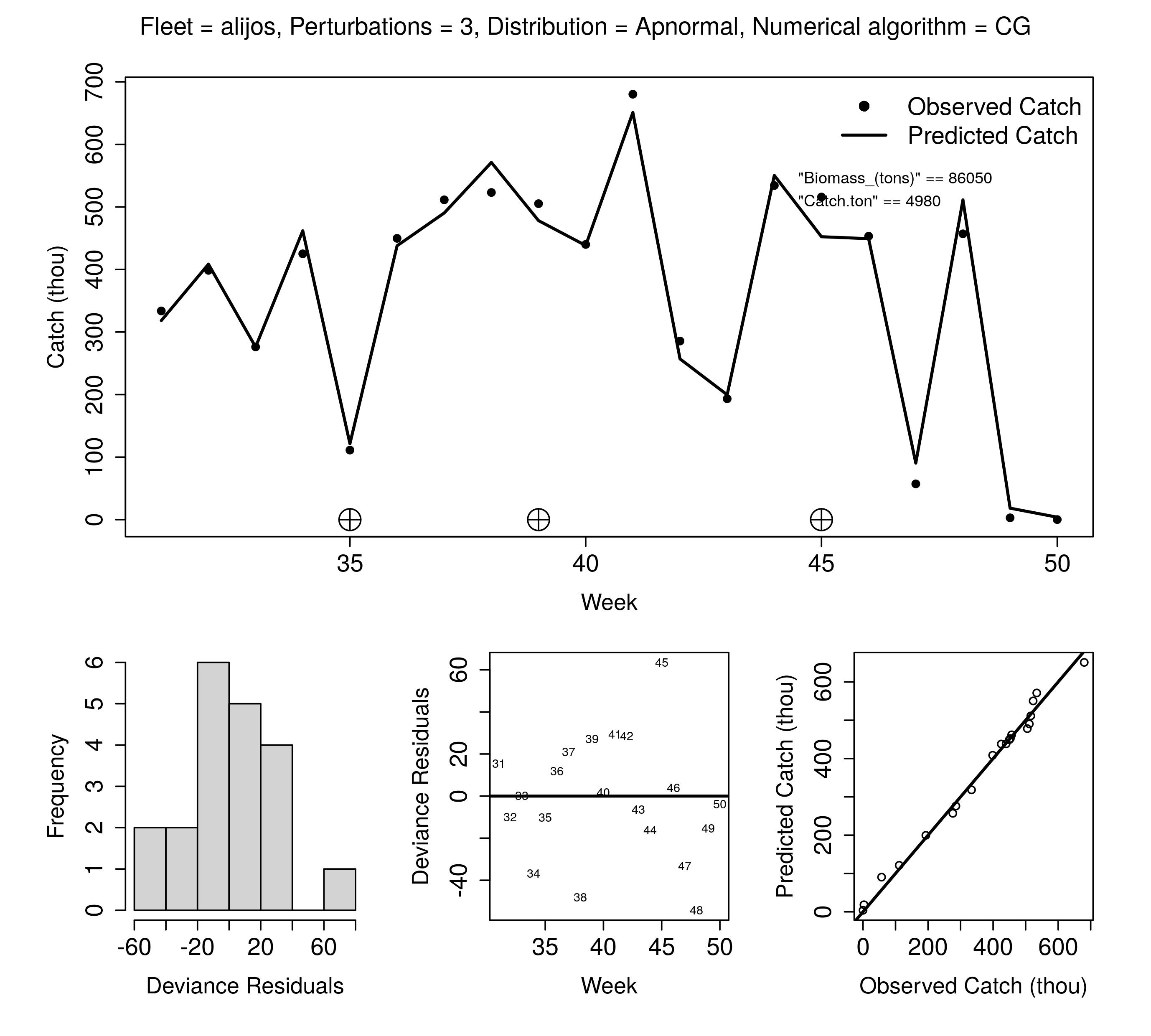

Supplement: S1 File — Model fit to data (top panel; dots: data; line: model) and residual diagnostics (three bottom panels; left: residual histogram; centre: residual cloud; right: quantile-quantile plot) for 22 fishing seasons of O. maya in Yucatan, Mexico. (ZIP) [file pone.0307836.s001.zip › FigS02CatDynMaya2001.jpg]

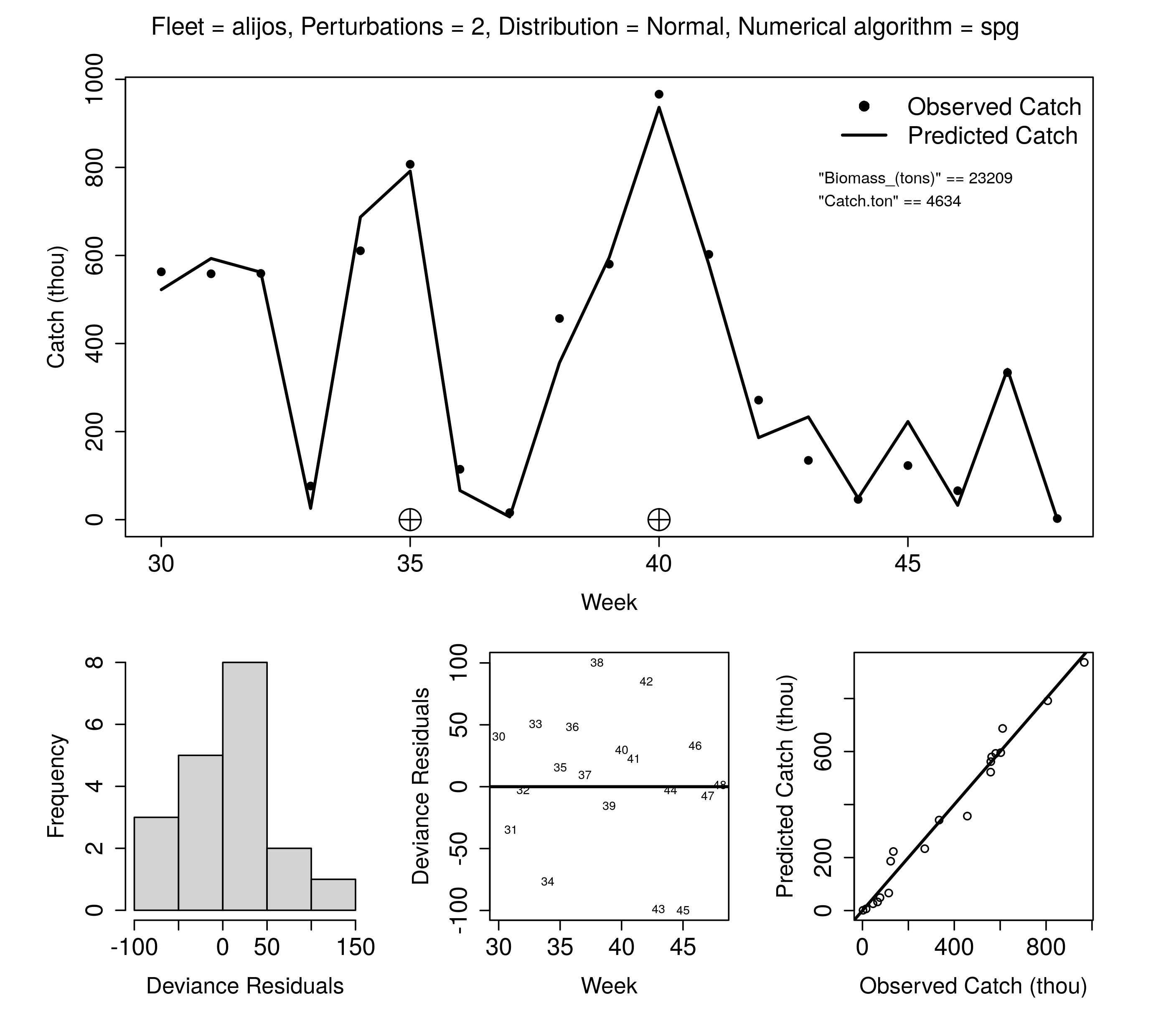

Supplement: S1 File — Model fit to data (top panel; dots: data; line: model) and residual diagnostics (three bottom panels; left: residual histogram; centre: residual cloud; right: quantile-quantile plot) for 22 fishing seasons of O. maya in Yucatan, Mexico. (ZIP) [file pone.0307836.s001.zip › FigS03CatDynMaya2002.jpg]

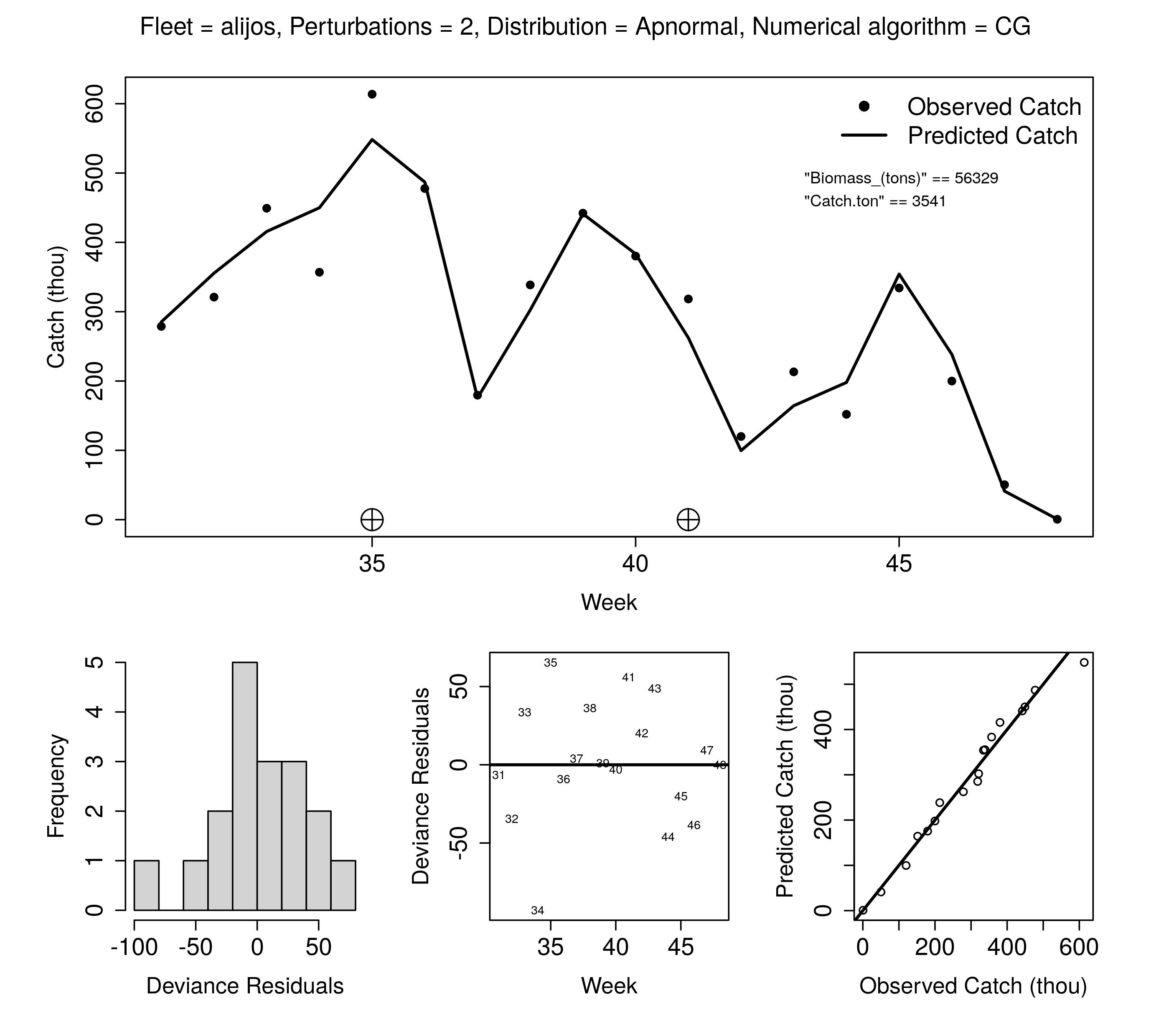

Supplement: S1 File — Model fit to data (top panel; dots: data; line: model) and residual diagnostics (three bottom panels; left: residual histogram; centre: residual cloud; right: quantile-quantile plot) for 22 fishing seasons of O. maya in Yucatan, Mexico. (ZIP) [file pone.0307836.s001.zip › FigS04CatDynMaya2003.jpg]

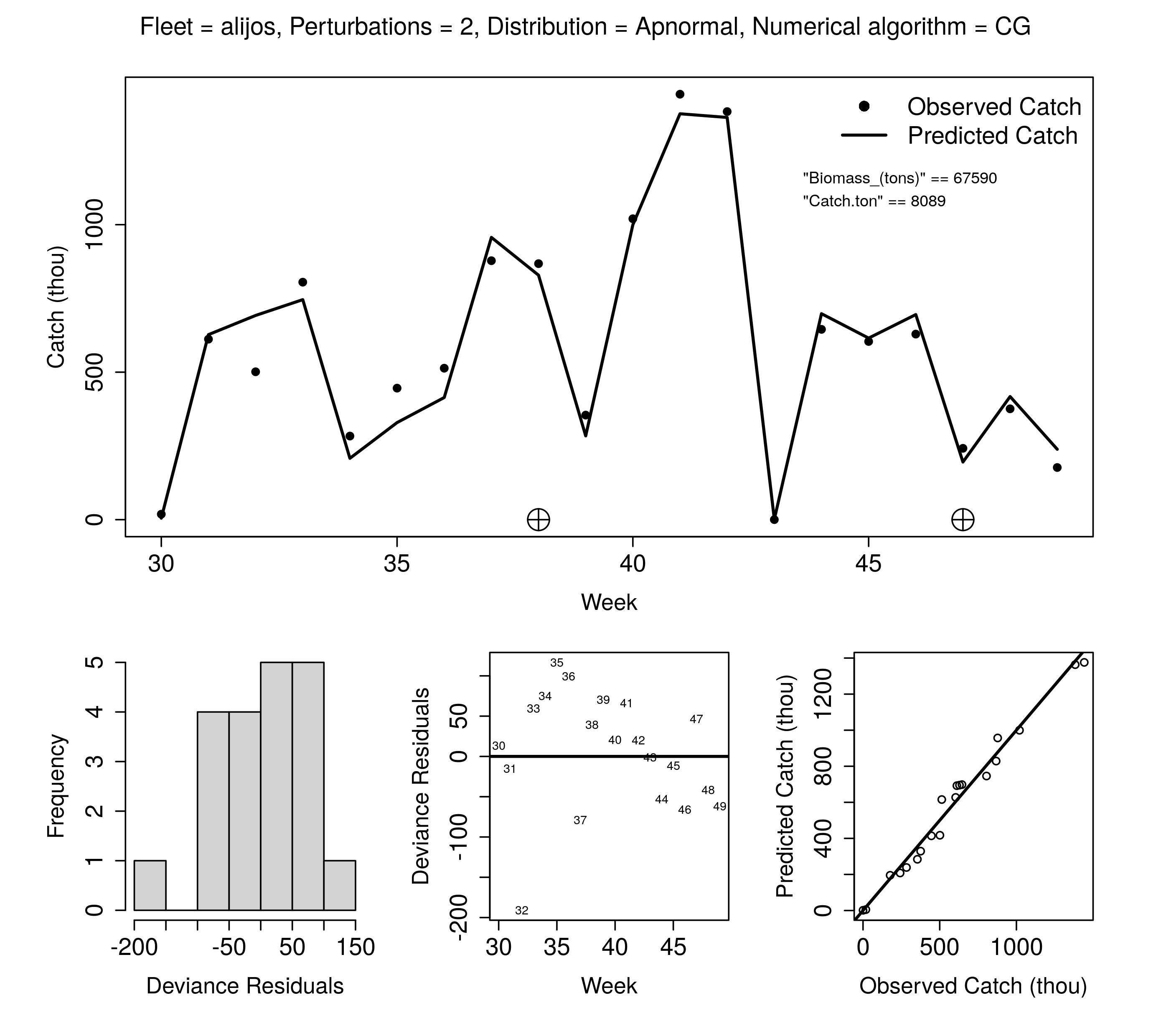

Supplement: S1 File — Model fit to data (top panel; dots: data; line: model) and residual diagnostics (three bottom panels; left: residual histogram; centre: residual cloud; right: quantile-quantile plot) for 22 fishing seasons of O. maya in Yucatan, Mexico. (ZIP) [file pone.0307836.s001.zip › FigS05CatDynMaya2004.jpg]

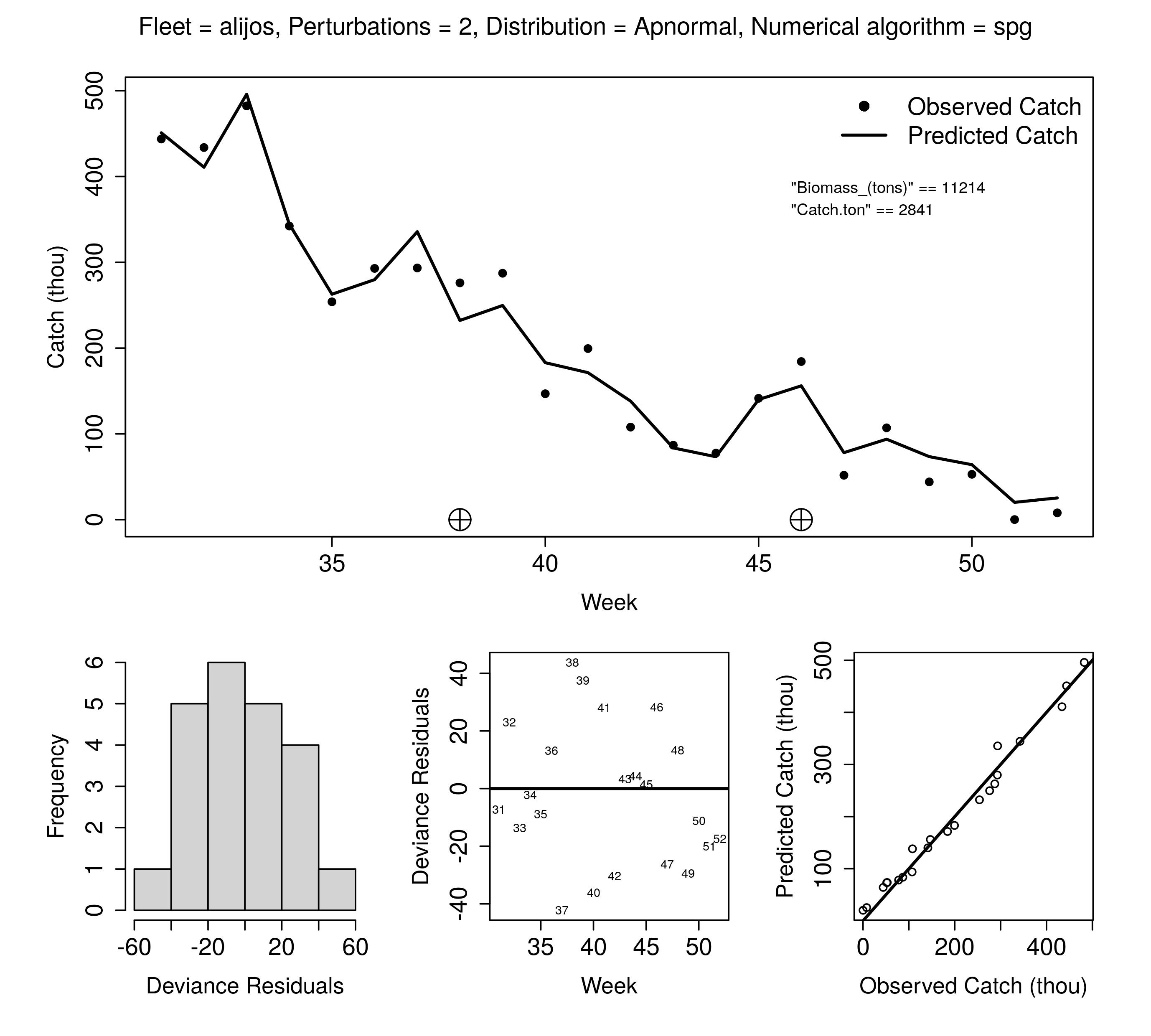

Supplement: S1 File — Model fit to data (top panel; dots: data; line: model) and residual diagnostics (three bottom panels; left: residual histogram; centre: residual cloud; right: quantile-quantile plot) for 22 fishing seasons of O. maya in Yucatan, Mexico. (ZIP) [file pone.0307836.s001.zip › FigS06CatDynMaya2005.jpg]

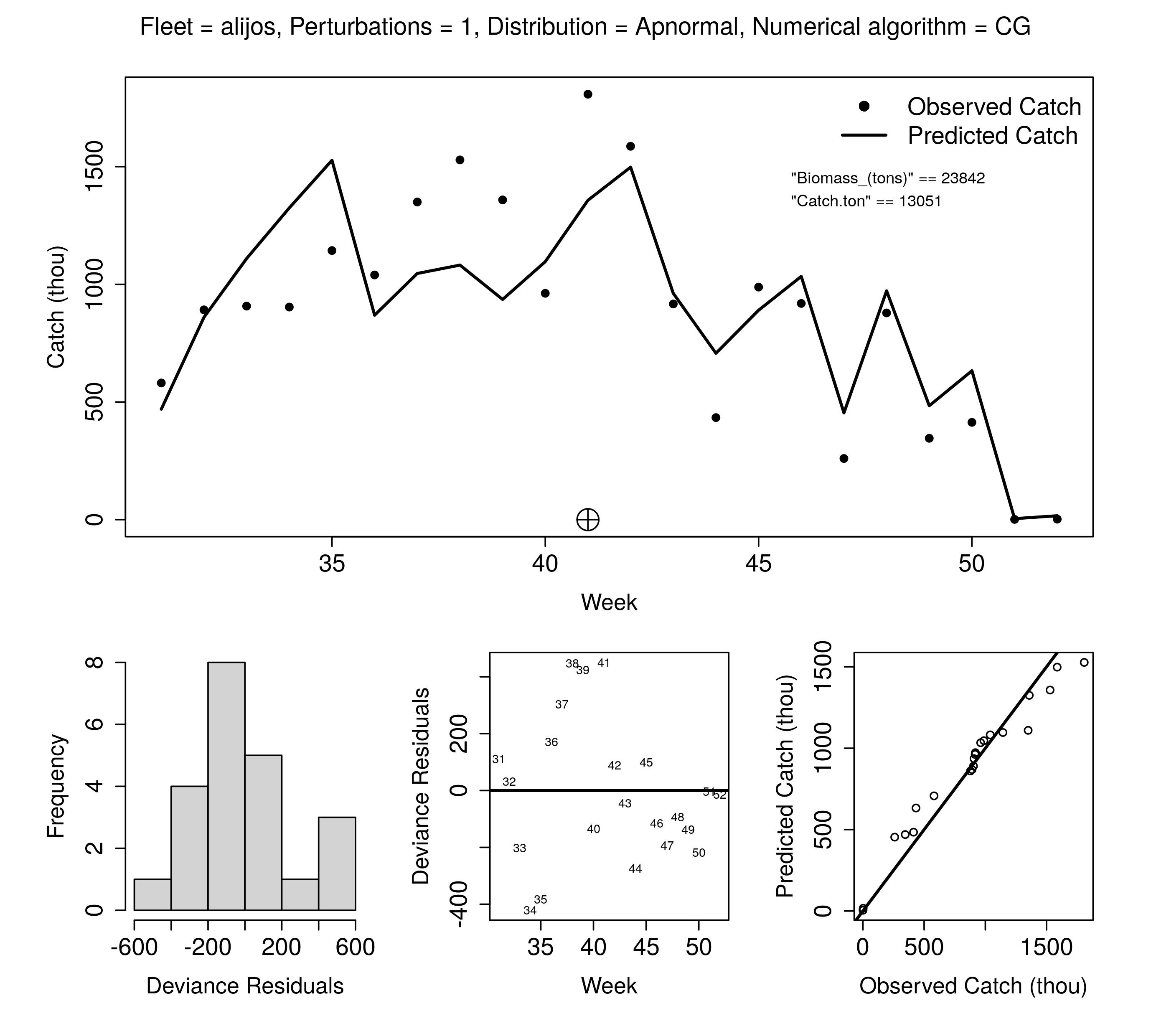

Supplement: S1 File — Model fit to data (top panel; dots: data; line: model) and residual diagnostics (three bottom panels; left: residual histogram; centre: residual cloud; right: quantile-quantile plot) for 22 fishing seasons of O. maya in Yucatan, Mexico. (ZIP) [file pone.0307836.s001.zip › FigS07CatDynMaya2006.jpg]

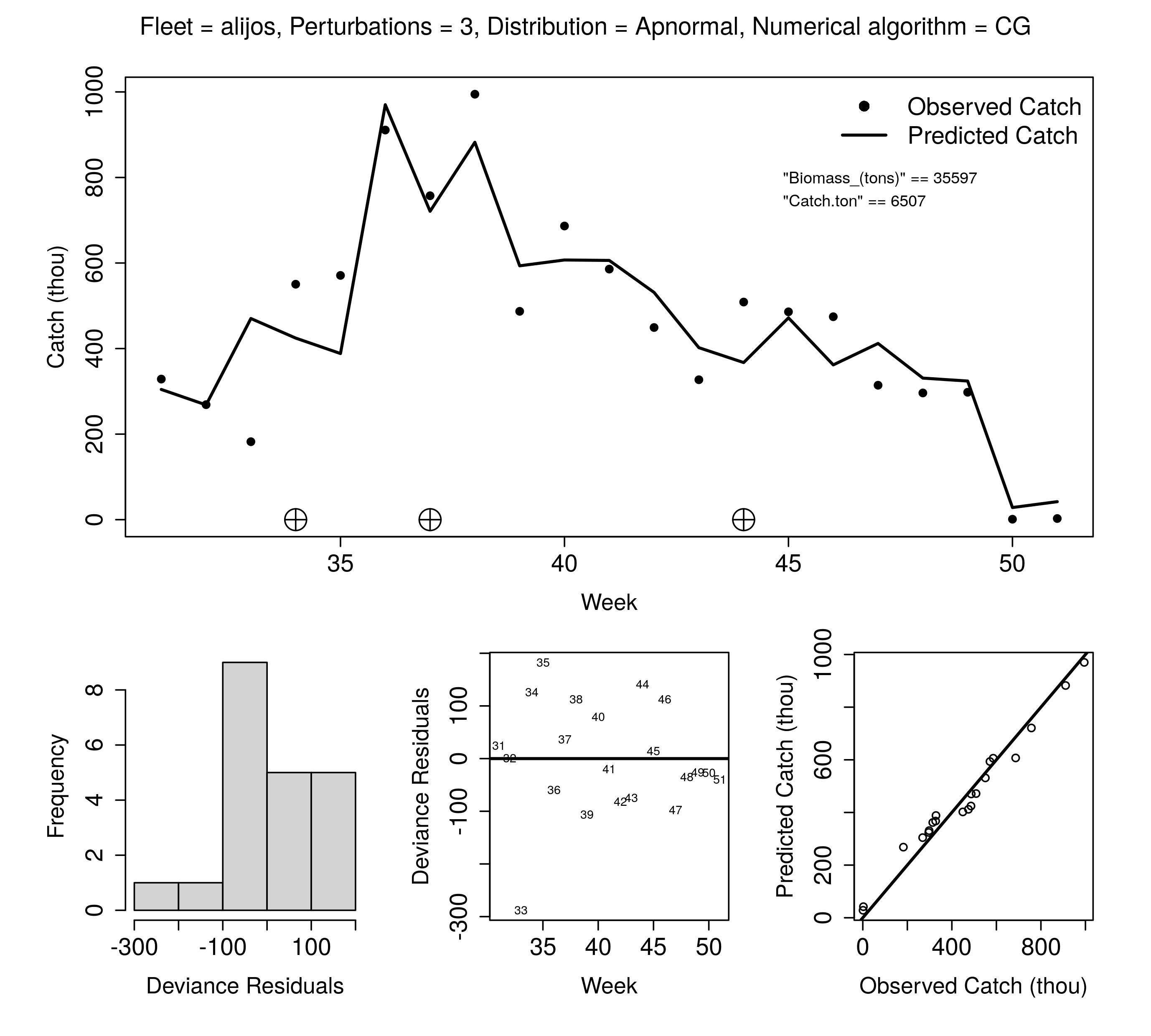

Supplement: S1 File — Model fit to data (top panel; dots: data; line: model) and residual diagnostics (three bottom panels; left: residual histogram; centre: residual cloud; right: quantile-quantile plot) for 22 fishing seasons of O. maya in Yucatan, Mexico. (ZIP) [file pone.0307836.s001.zip › FigS08CatDynMaya2007.jpg]

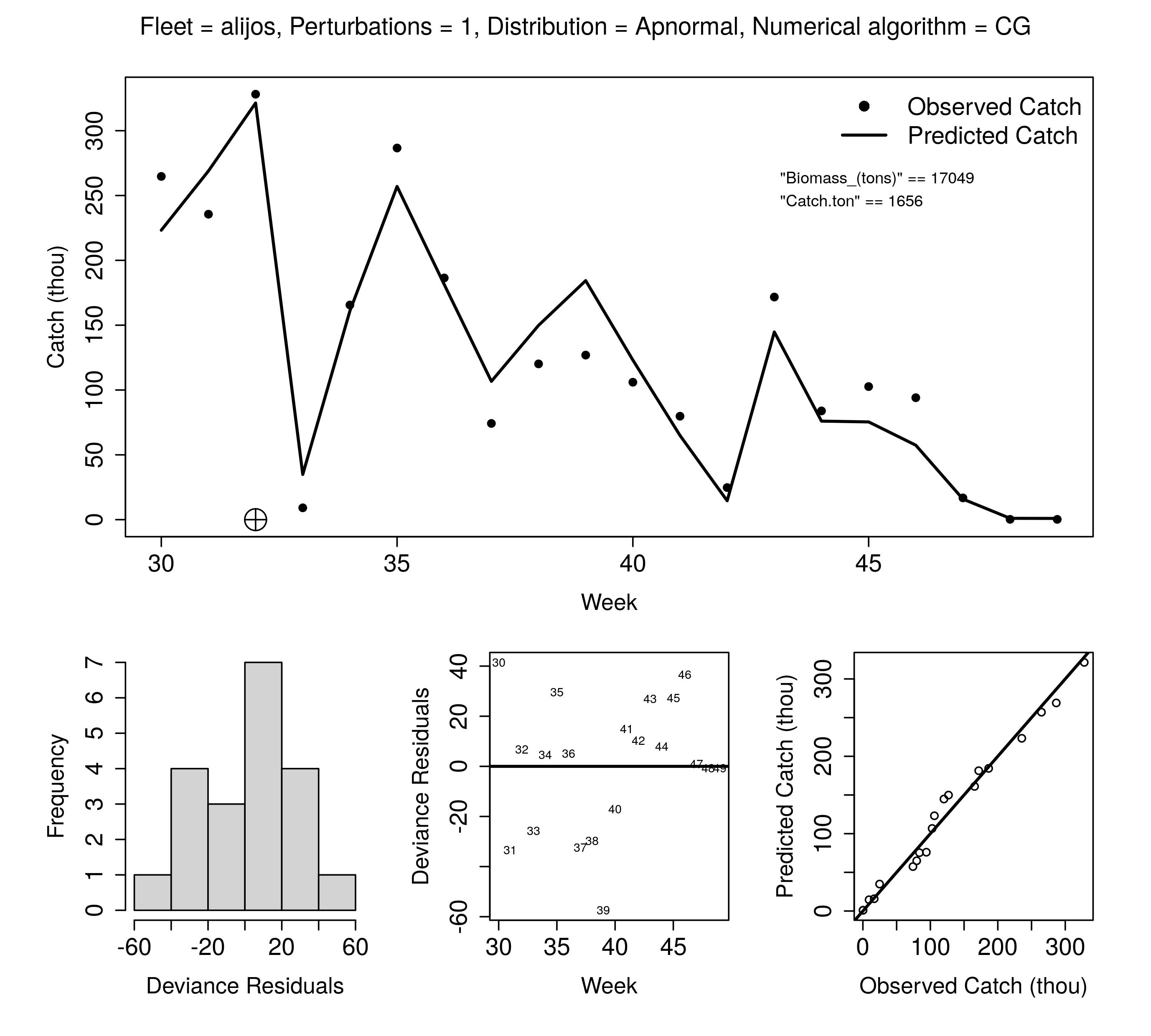

Supplement: S1 File — Model fit to data (top panel; dots: data; line: model) and residual diagnostics (three bottom panels; left: residual histogram; centre: residual cloud; right: quantile-quantile plot) for 22 fishing seasons of O. maya in Yucatan, Mexico. (ZIP) [file pone.0307836.s001.zip › FigS09CatDynMaya2008.jpg]

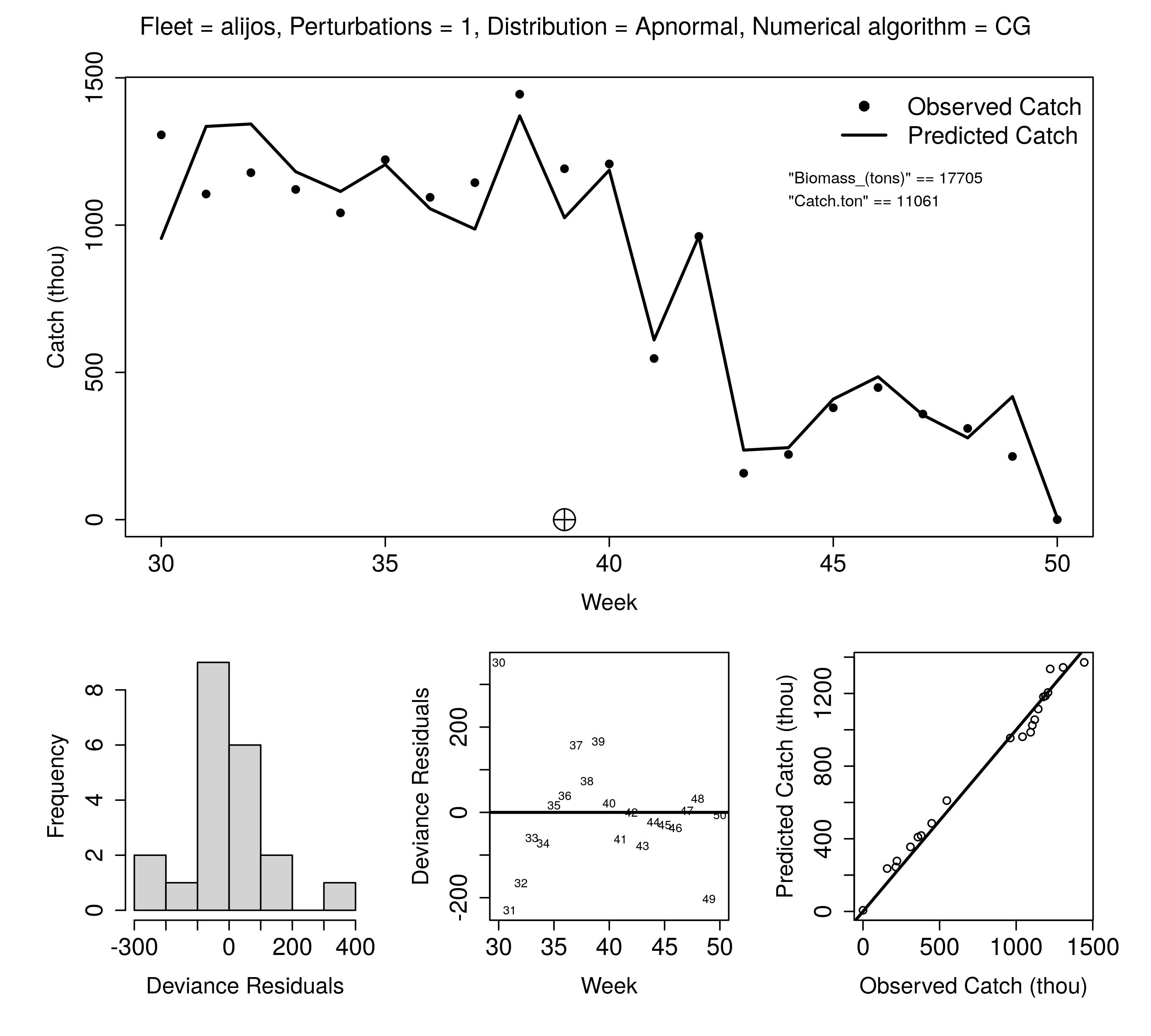

Supplement: S1 File — Model fit to data (top panel; dots: data; line: model) and residual diagnostics (three bottom panels; left: residual histogram; centre: residual cloud; right: quantile-quantile plot) for 22 fishing seasons of O. maya in Yucatan, Mexico. (ZIP) [file pone.0307836.s001.zip › FigS10CatDynMaya2009.jpg]

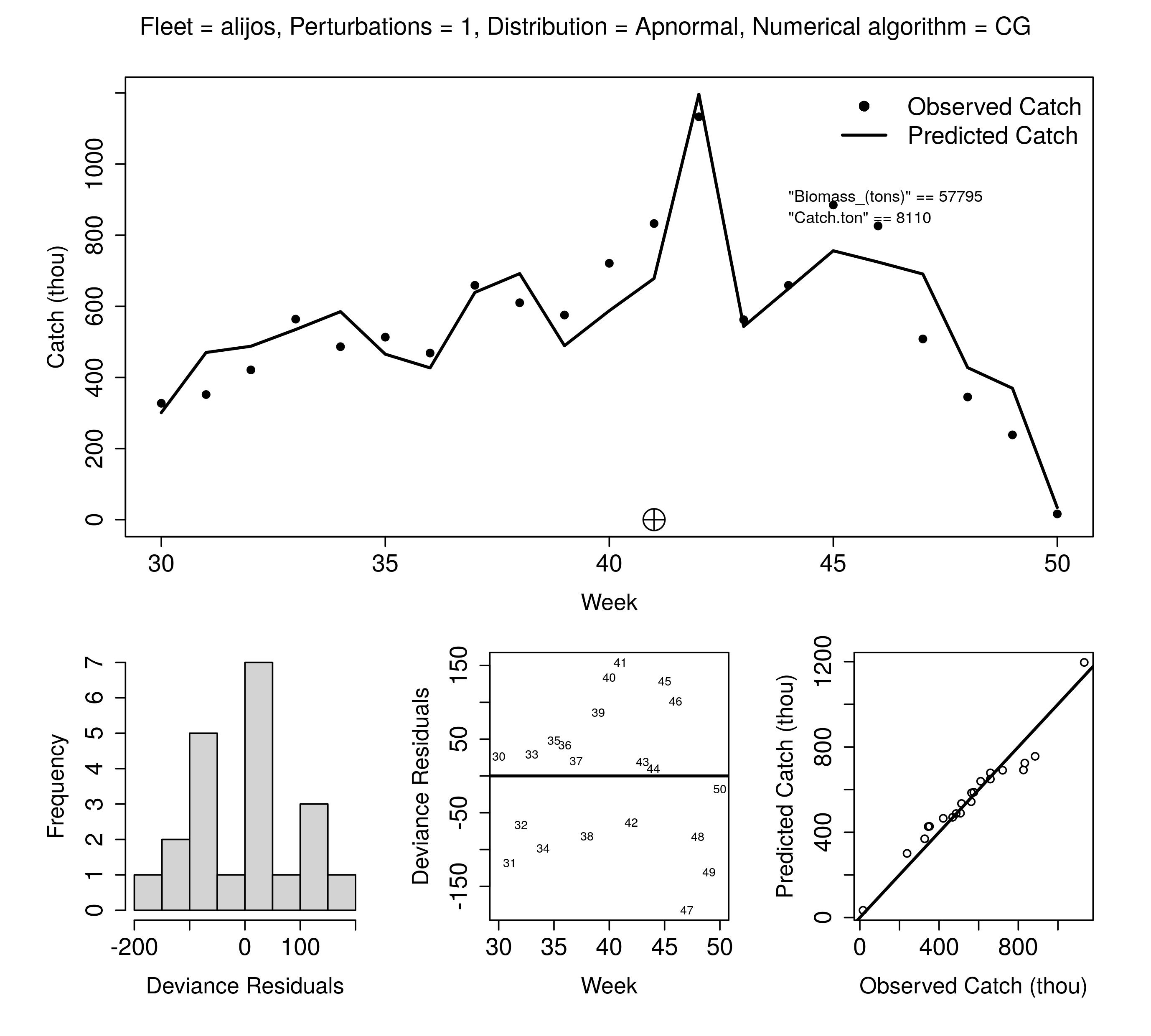

Supplement: S1 File — Model fit to data (top panel; dots: data; line: model) and residual diagnostics (three bottom panels; left: residual histogram; centre: residual cloud; right: quantile-quantile plot) for 22 fishing seasons of O. maya in Yucatan, Mexico. (ZIP) [file pone.0307836.s001.zip › FigS11CatDynMaya2010.jpg]

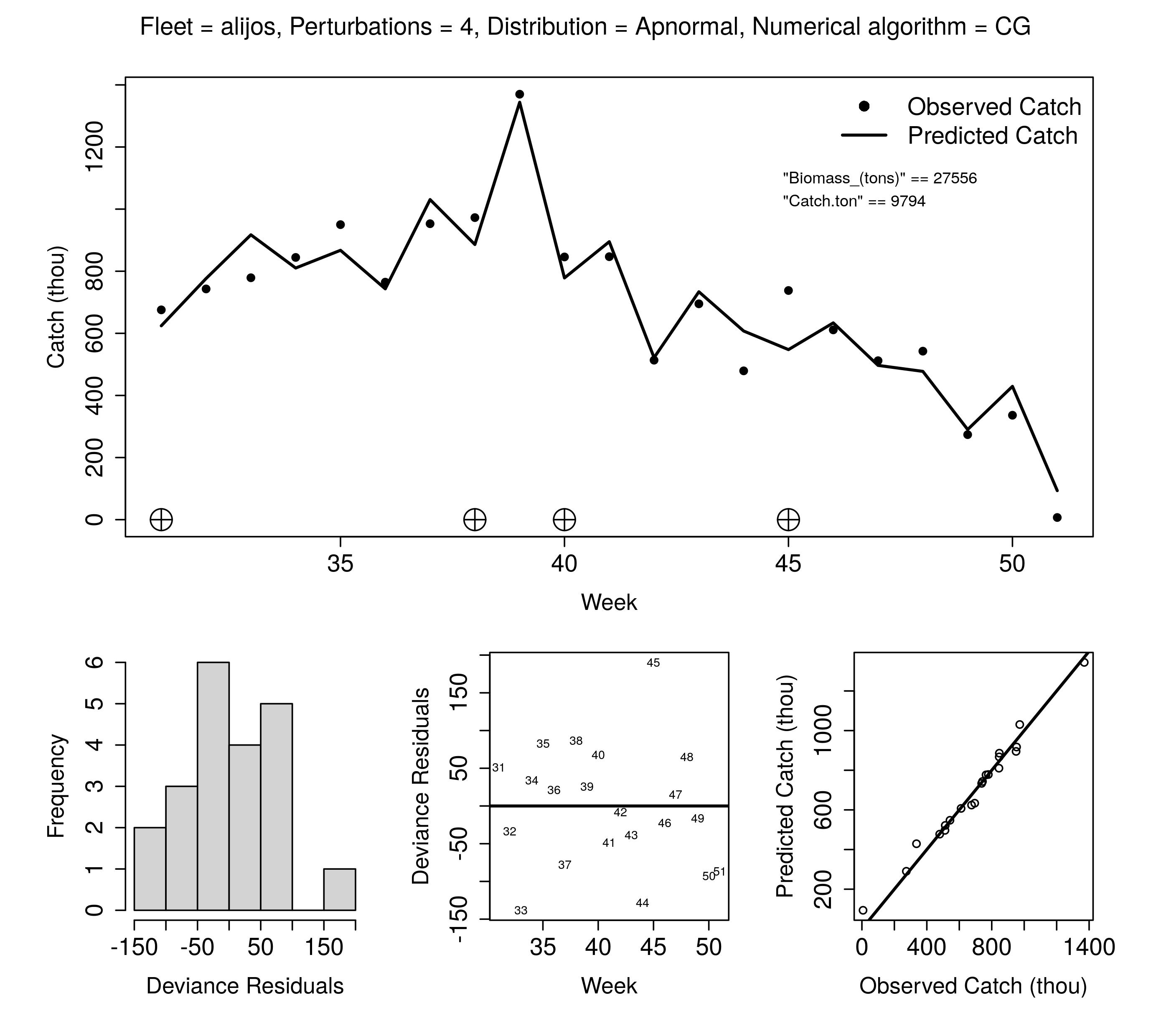

Supplement: S1 File — Model fit to data (top panel; dots: data; line: model) and residual diagnostics (three bottom panels; left: residual histogram; centre: residual cloud; right: quantile-quantile plot) for 22 fishing seasons of O. maya in Yucatan, Mexico. (ZIP) [file pone.0307836.s001.zip › FigS12CatDynMaya2011.jpg]

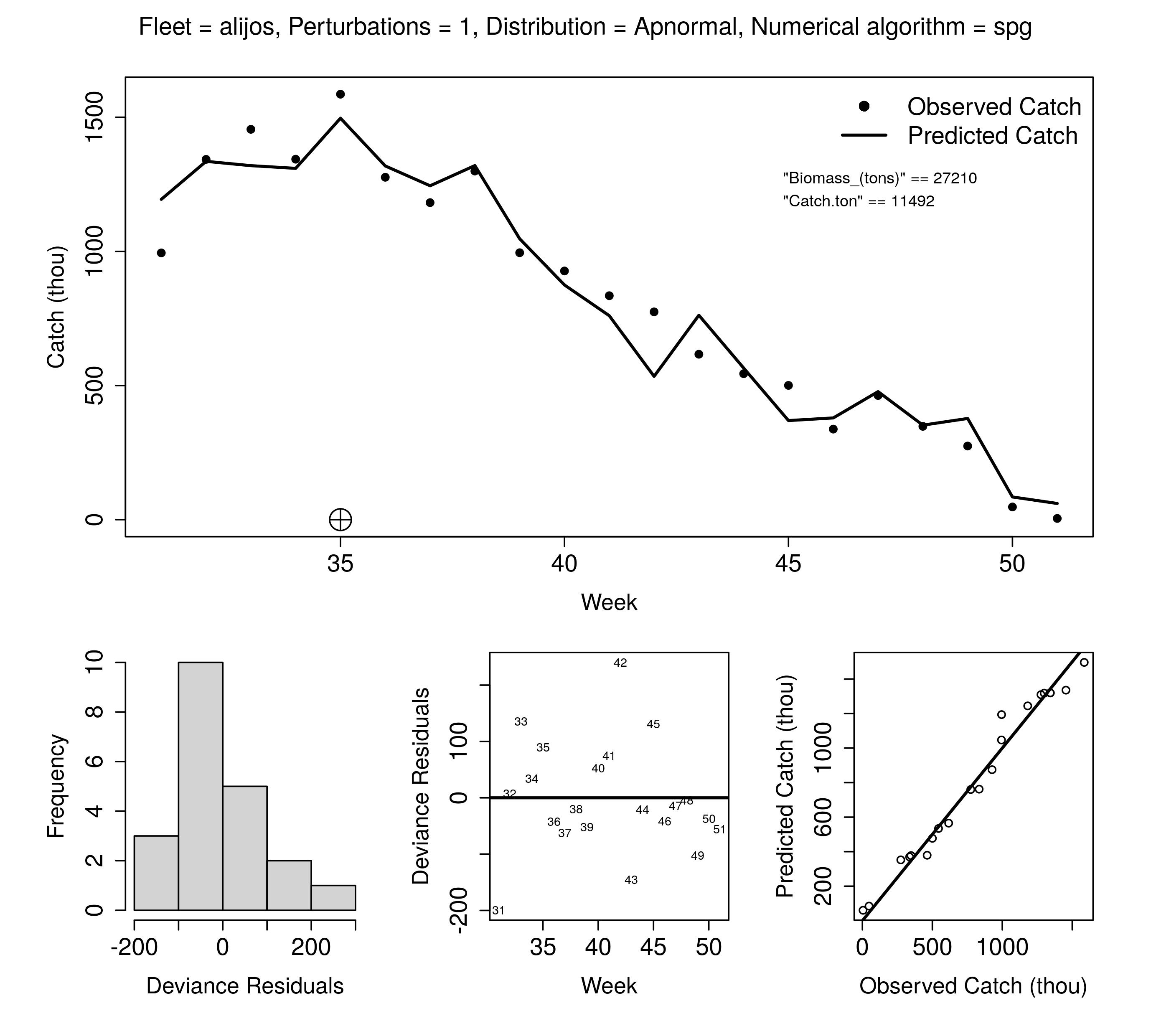

Supplement: S1 File — Model fit to data (top panel; dots: data; line: model) and residual diagnostics (three bottom panels; left: residual histogram; centre: residual cloud; right: quantile-quantile plot) for 22 fishing seasons of O. maya in Yucatan, Mexico. (ZIP) [file pone.0307836.s001.zip › FigS13CatDynMaya2012.jpg]

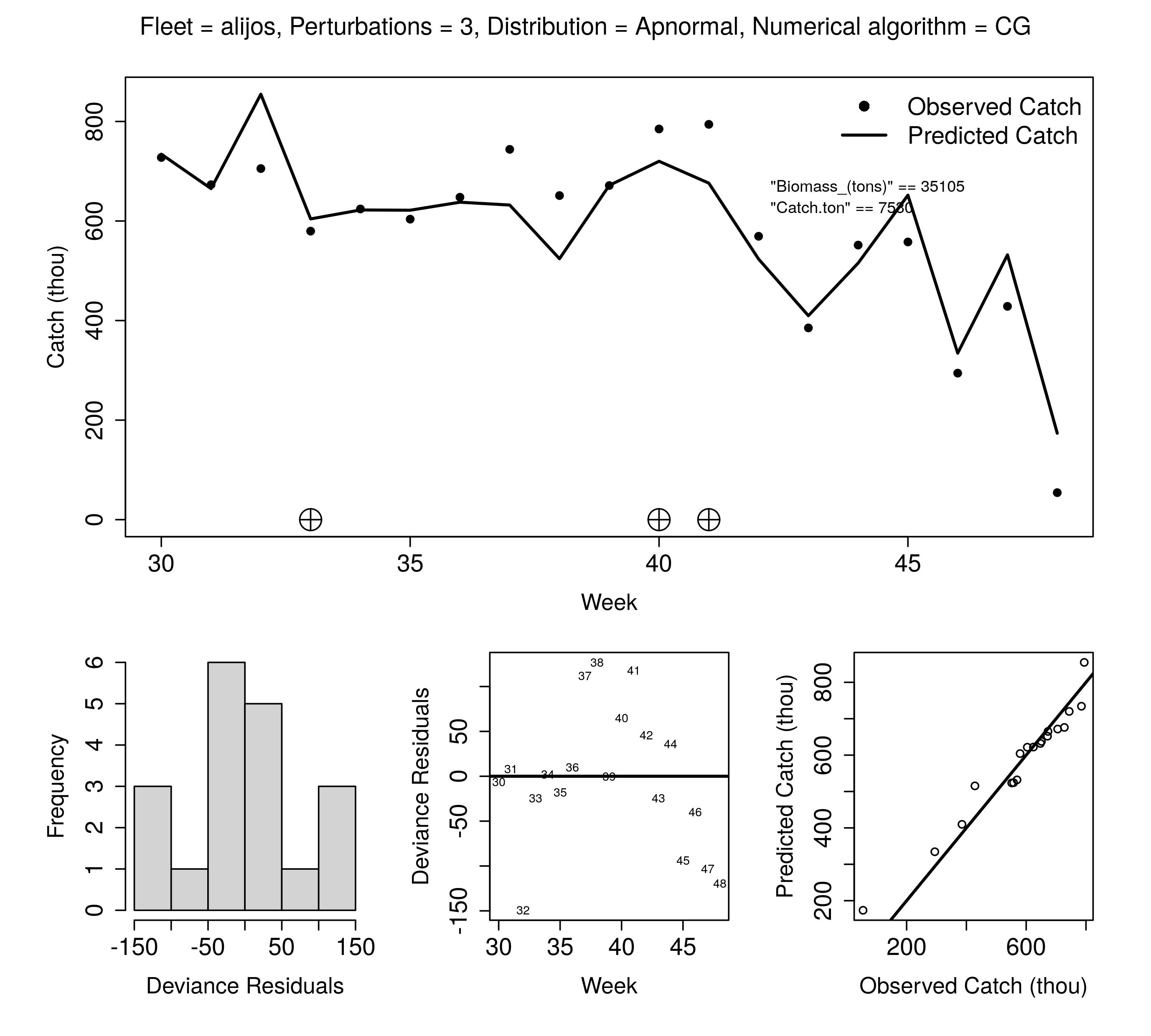

Supplement: S1 File — Model fit to data (top panel; dots: data; line: model) and residual diagnostics (three bottom panels; left: residual histogram; centre: residual cloud; right: quantile-quantile plot) for 22 fishing seasons of O. maya in Yucatan, Mexico. (ZIP) [file pone.0307836.s001.zip › FigS14CatDynMaya2013.jpg]

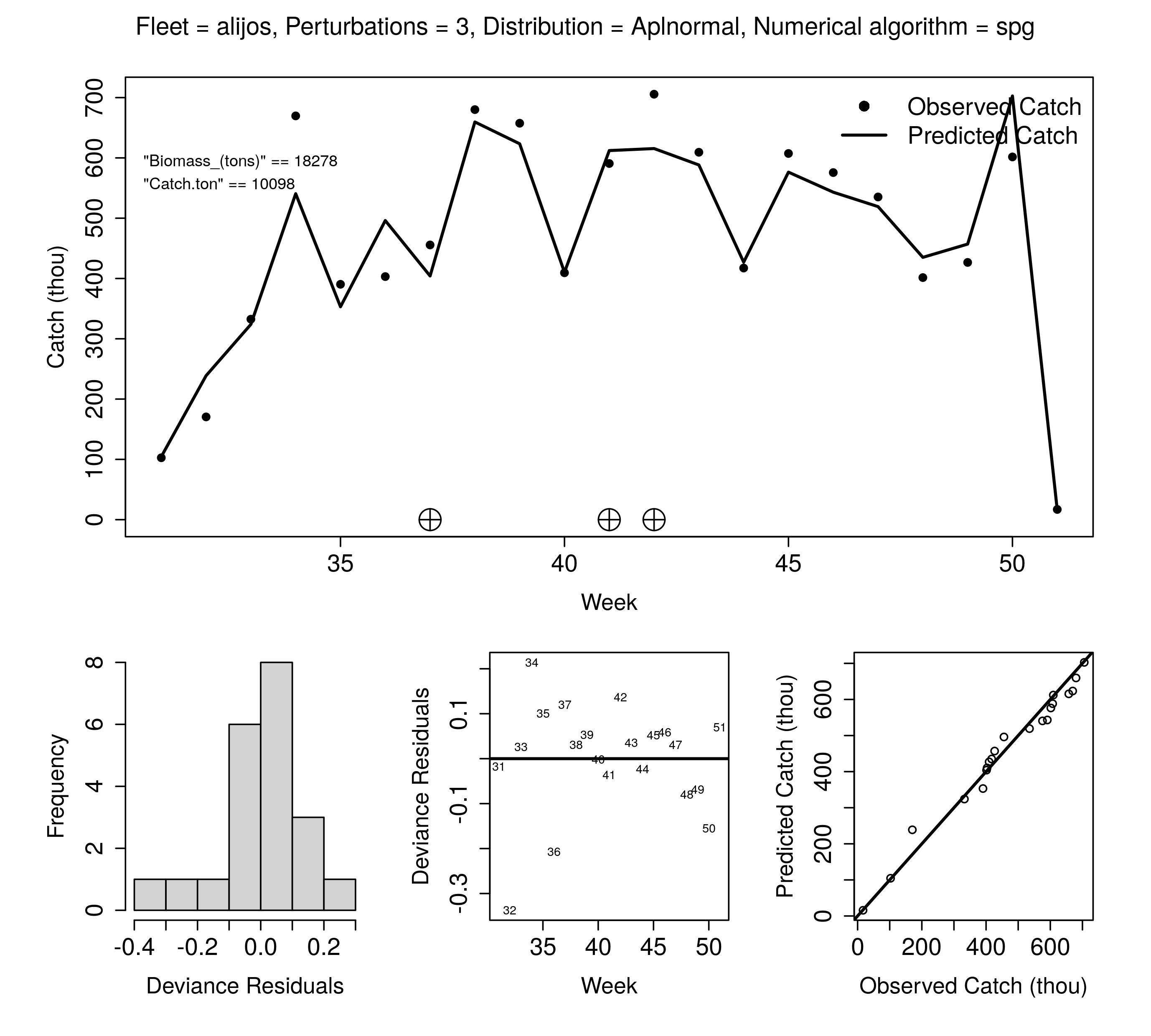

Supplement: S2 File — Model fit to data (top panel; dots: data; line: model) and residual diagnostics (three bottom panels; left: residual histogram; centre: residual cloud; right: quantile-quantile plot) for 22 fishing seasons of O. americanus in Yucatan, Mexico. (ZIP) [file pone.0307836.s002.zip › FigS39CatDynAmer2016.jpg]

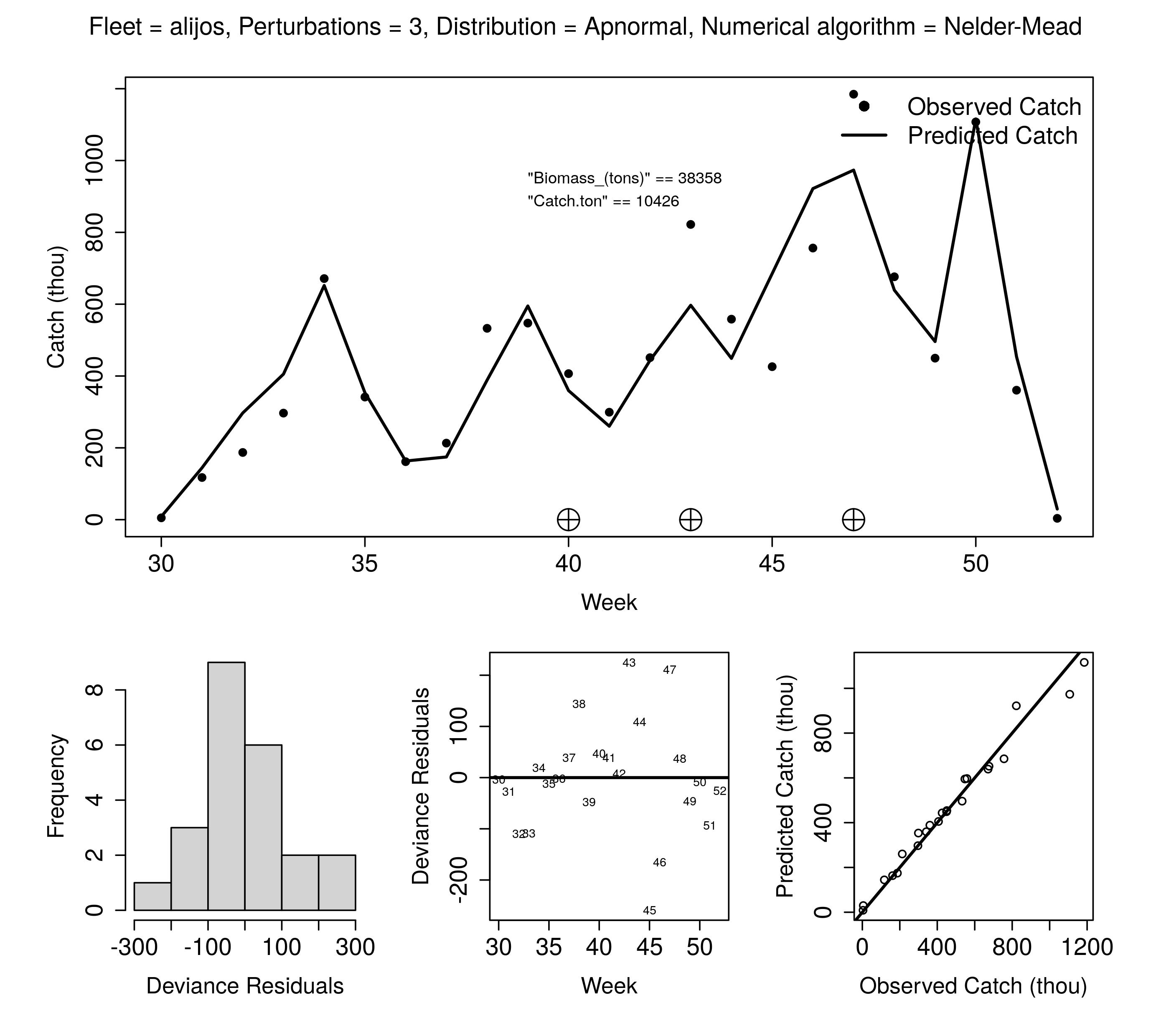

Supplement: S2 File — Model fit to data (top panel; dots: data; line: model) and residual diagnostics (three bottom panels; left: residual histogram; centre: residual cloud; right: quantile-quantile plot) for 22 fishing seasons of O. americanus in Yucatan, Mexico. (ZIP) [file pone.0307836.s002.zip › FigS40CatDynAmer2017.jpg]

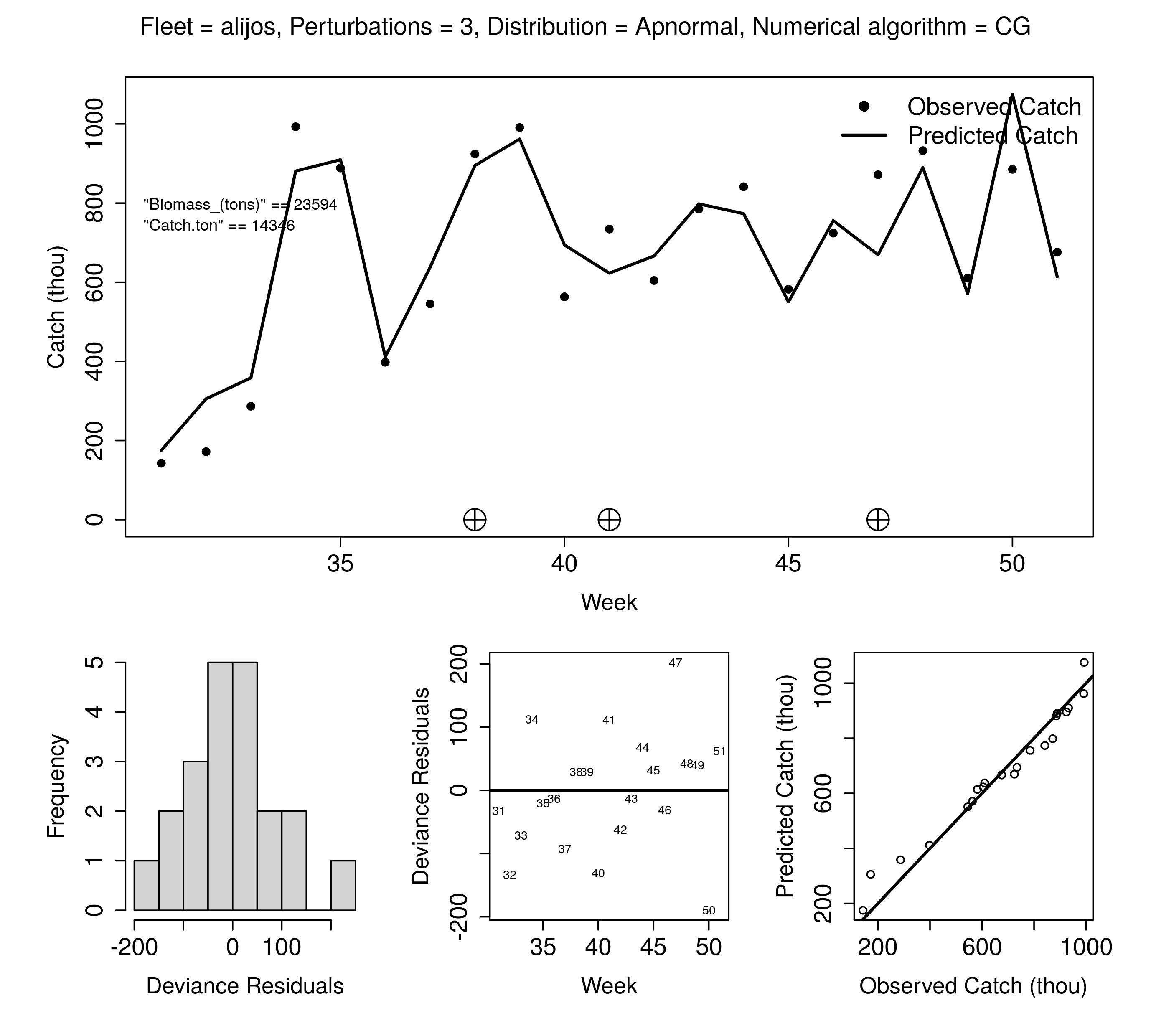

Supplement: S2 File — Model fit to data (top panel; dots: data; line: model) and residual diagnostics (three bottom panels; left: residual histogram; centre: residual cloud; right: quantile-quantile plot) for 22 fishing seasons of O. americanus in Yucatan, Mexico. (ZIP) [file pone.0307836.s002.zip › FigS41CatDynAmer2018.jpg]

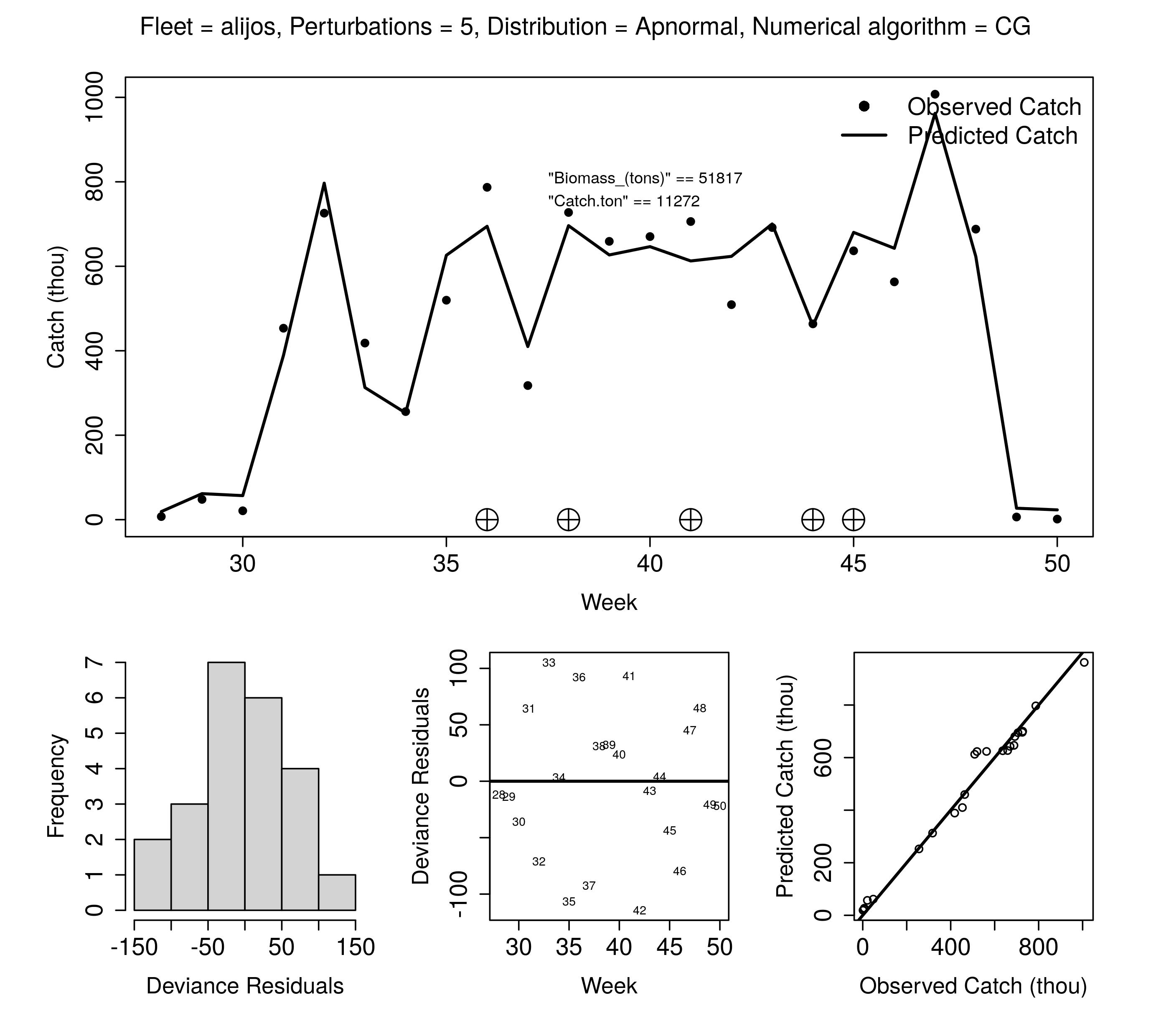

Supplement: S2 File — Model fit to data (top panel; dots: data; line: model) and residual diagnostics (three bottom panels; left: residual histogram; centre: residual cloud; right: quantile-quantile plot) for 22 fishing seasons of O. americanus in Yucatan, Mexico. (ZIP) [file pone.0307836.s002.zip › FigS42CatDynAmer2019.jpg]

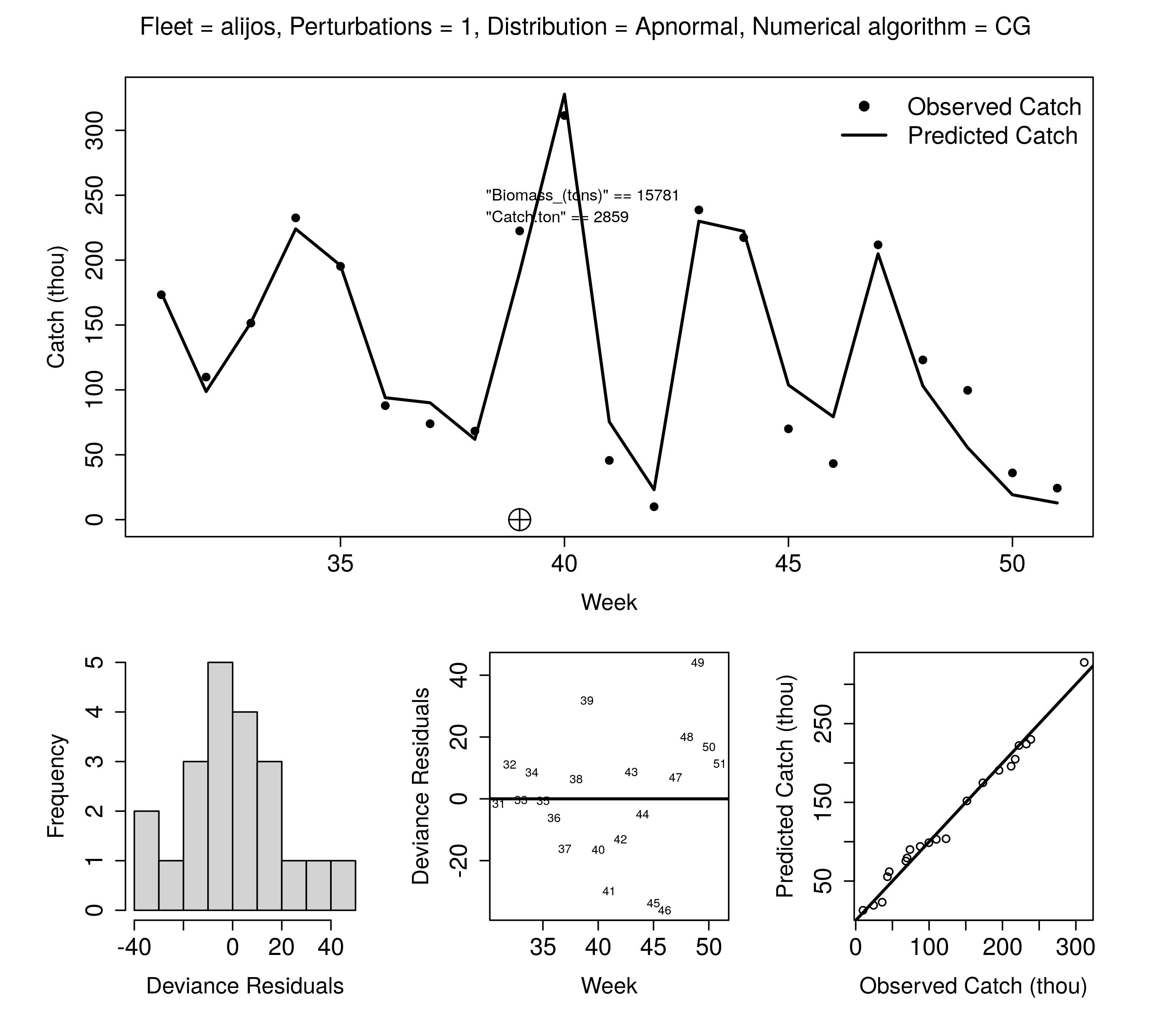

Supplement: S2 File — Model fit to data (top panel; dots: data; line: model) and residual diagnostics (three bottom panels; left: residual histogram; centre: residual cloud; right: quantile-quantile plot) for 22 fishing seasons of O. americanus in Yucatan, Mexico. (ZIP) [file pone.0307836.s002.zip › FigS43CatDynAmer2020.jpg]

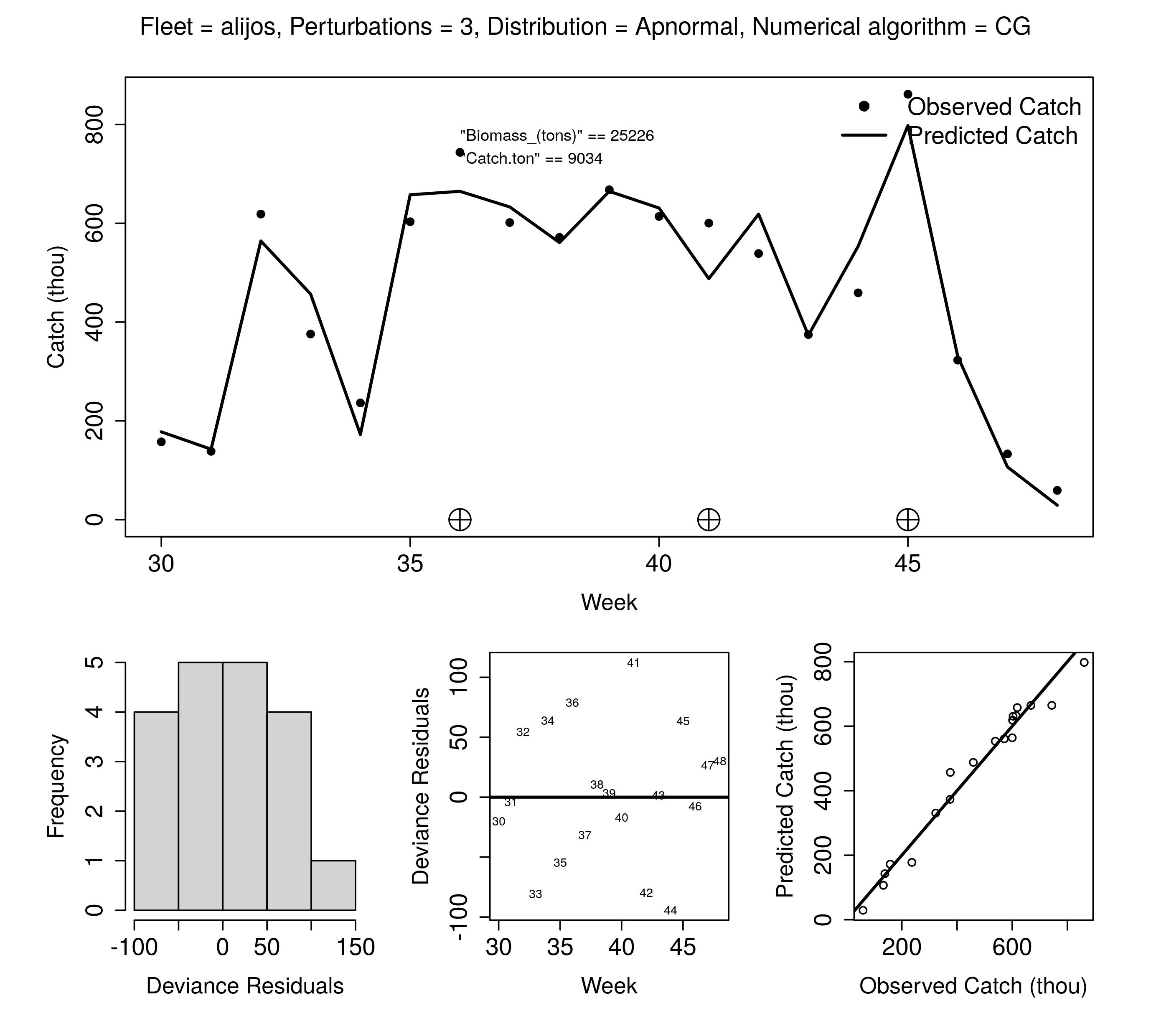

Supplement: S2 File — Model fit to data (top panel; dots: data; line: model) and residual diagnostics (three bottom panels; left: residual histogram; centre: residual cloud; right: quantile-quantile plot) for 22 fishing seasons of O. americanus in Yucatan, Mexico. (ZIP) [file pone.0307836.s002.zip › FigS44CatDynAmer2021.jpg]

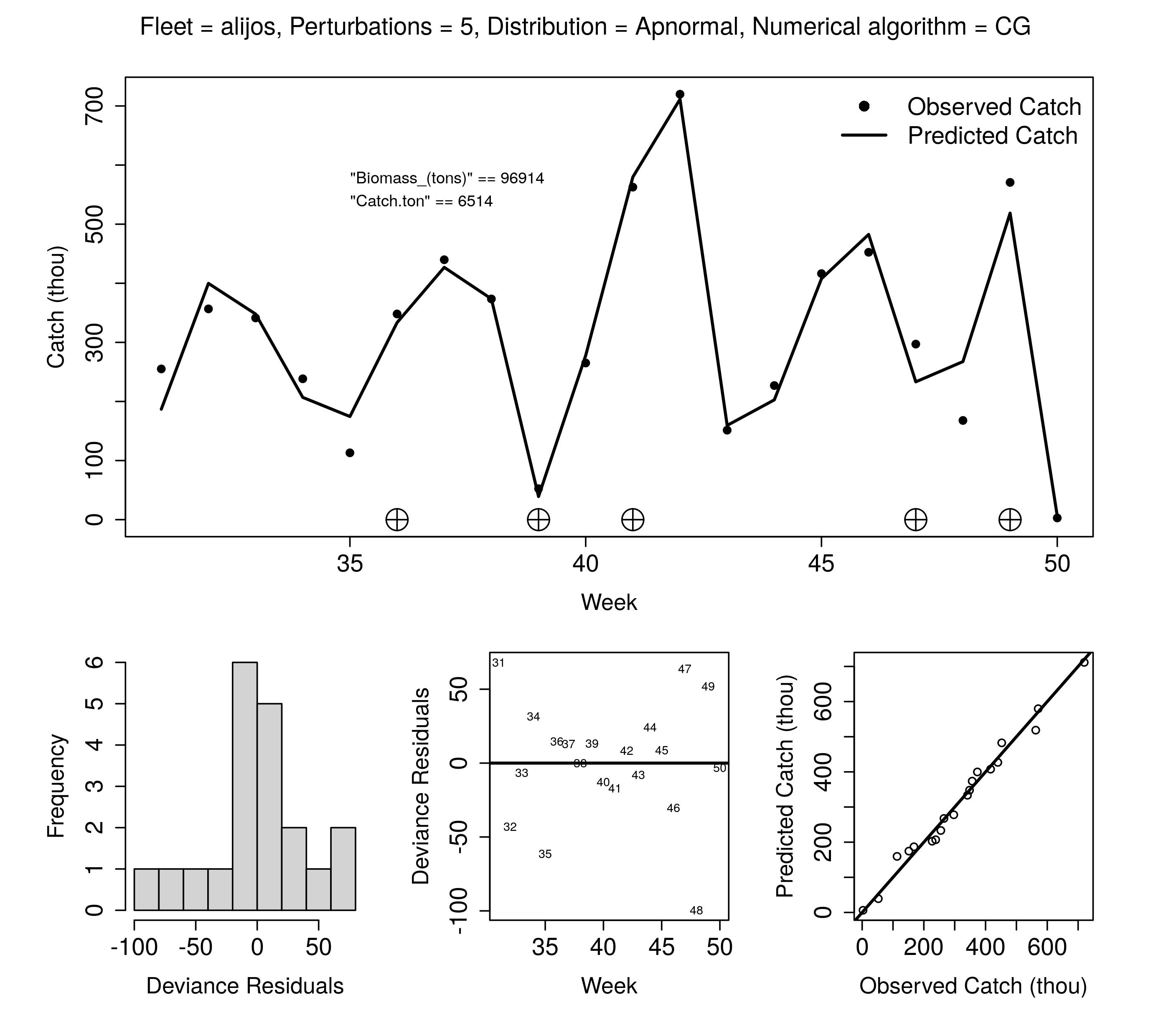

Supplement: S2 File — Model fit to data (top panel; dots: data; line: model) and residual diagnostics (three bottom panels; left: residual histogram; centre: residual cloud; right: quantile-quantile plot) for 22 fishing seasons of O. americanus in Yucatan, Mexico. (ZIP) [file pone.0307836.s002.zip › FigS23CatDynAmer2000.jpg]

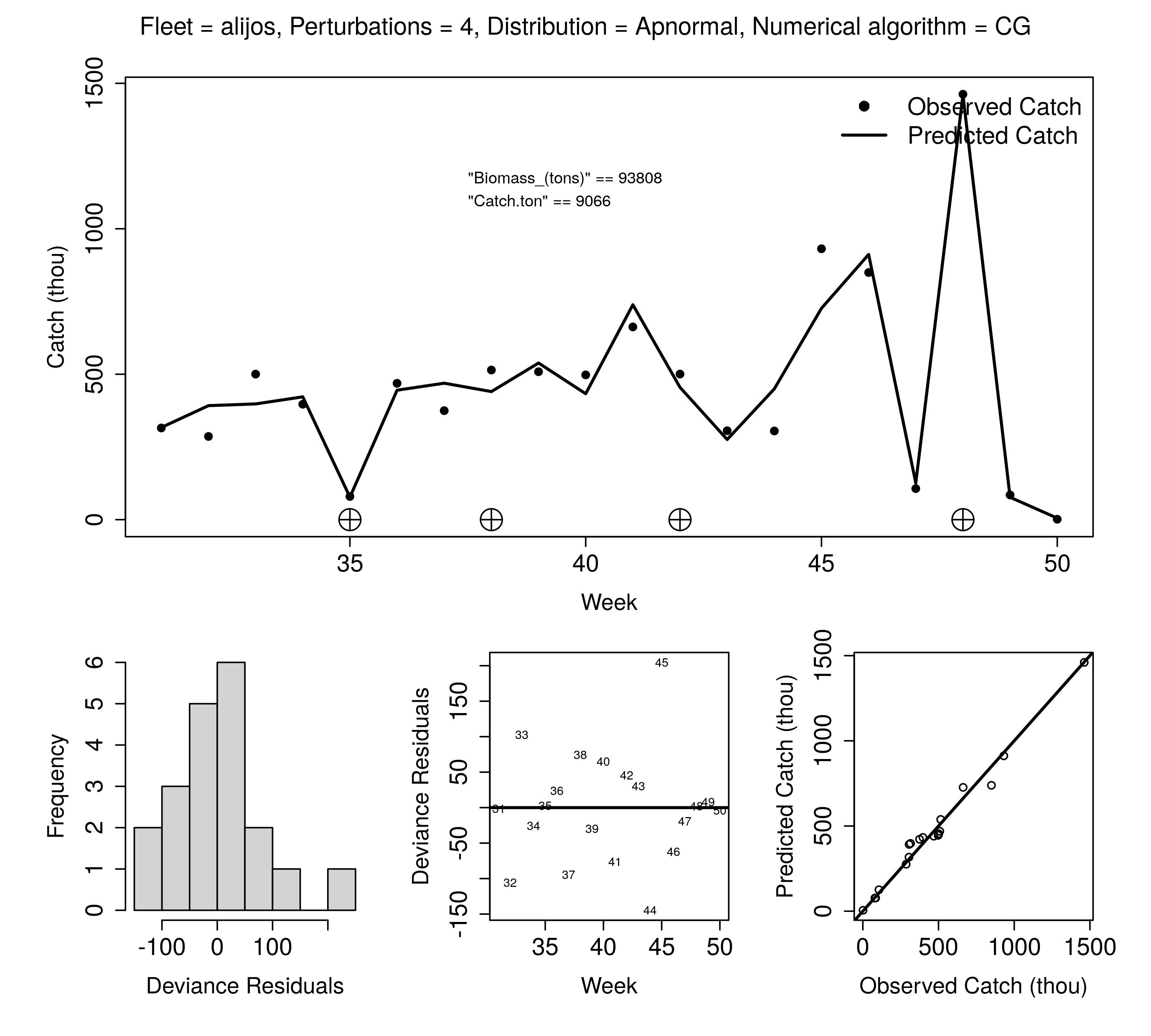

Supplement: S2 File — Model fit to data (top panel; dots: data; line: model) and residual diagnostics (three bottom panels; left: residual histogram; centre: residual cloud; right: quantile-quantile plot) for 22 fishing seasons of O. americanus in Yucatan, Mexico. (ZIP) [file pone.0307836.s002.zip › FigS24CatDynAmer2001.jpg]

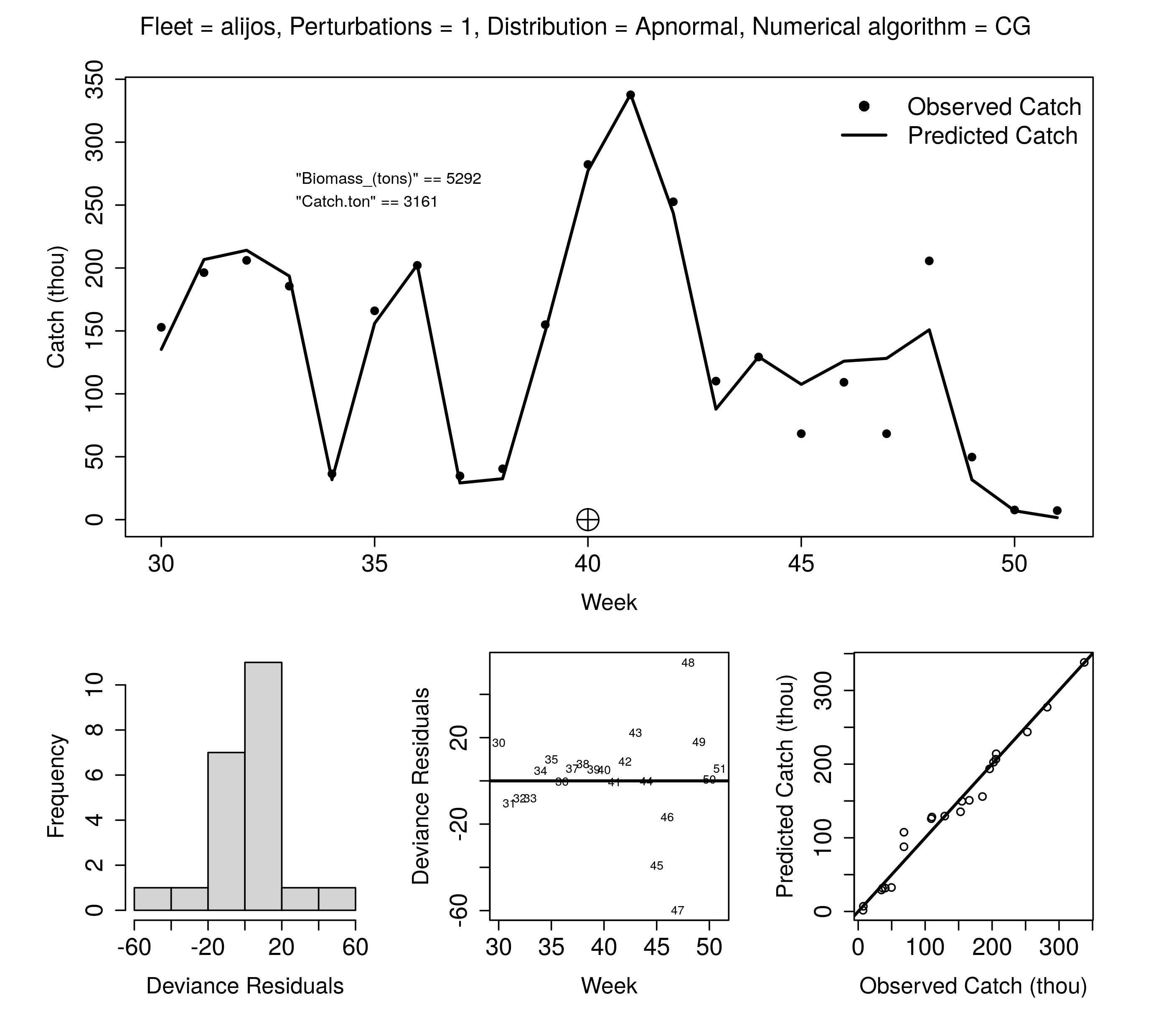

Supplement: S2 File — Model fit to data (top panel; dots: data; line: model) and residual diagnostics (three bottom panels; left: residual histogram; centre: residual cloud; right: quantile-quantile plot) for 22 fishing seasons of O. americanus in Yucatan, Mexico. (ZIP) [file pone.0307836.s002.zip › FigS25CatDynAmer2002.jpg]

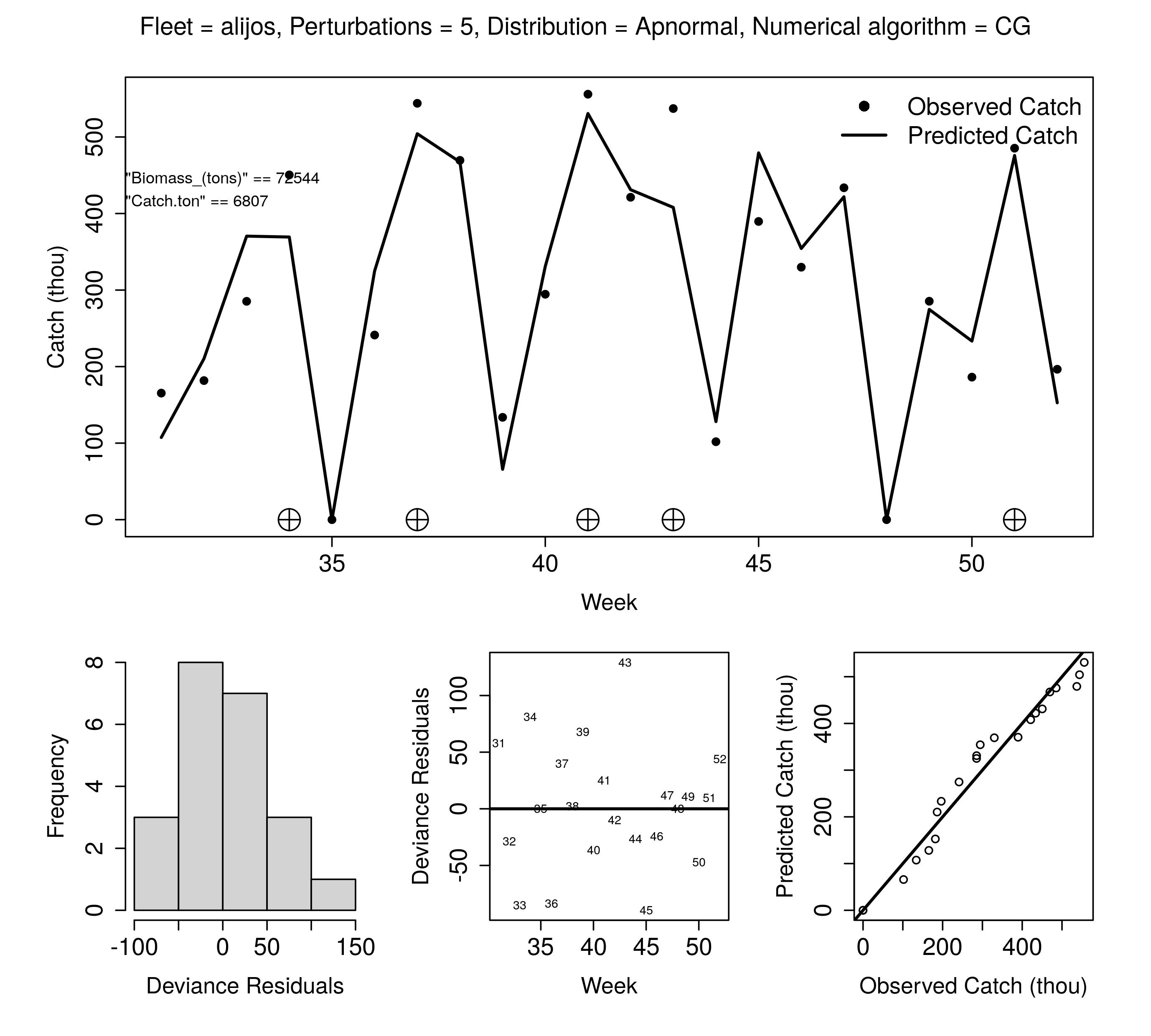

Supplement: S2 File — Model fit to data (top panel; dots: data; line: model) and residual diagnostics (three bottom panels; left: residual histogram; centre: residual cloud; right: quantile-quantile plot) for 22 fishing seasons of O. americanus in Yucatan, Mexico. (ZIP) [file pone.0307836.s002.zip › FigS26CatDynAmer2003.jpg]

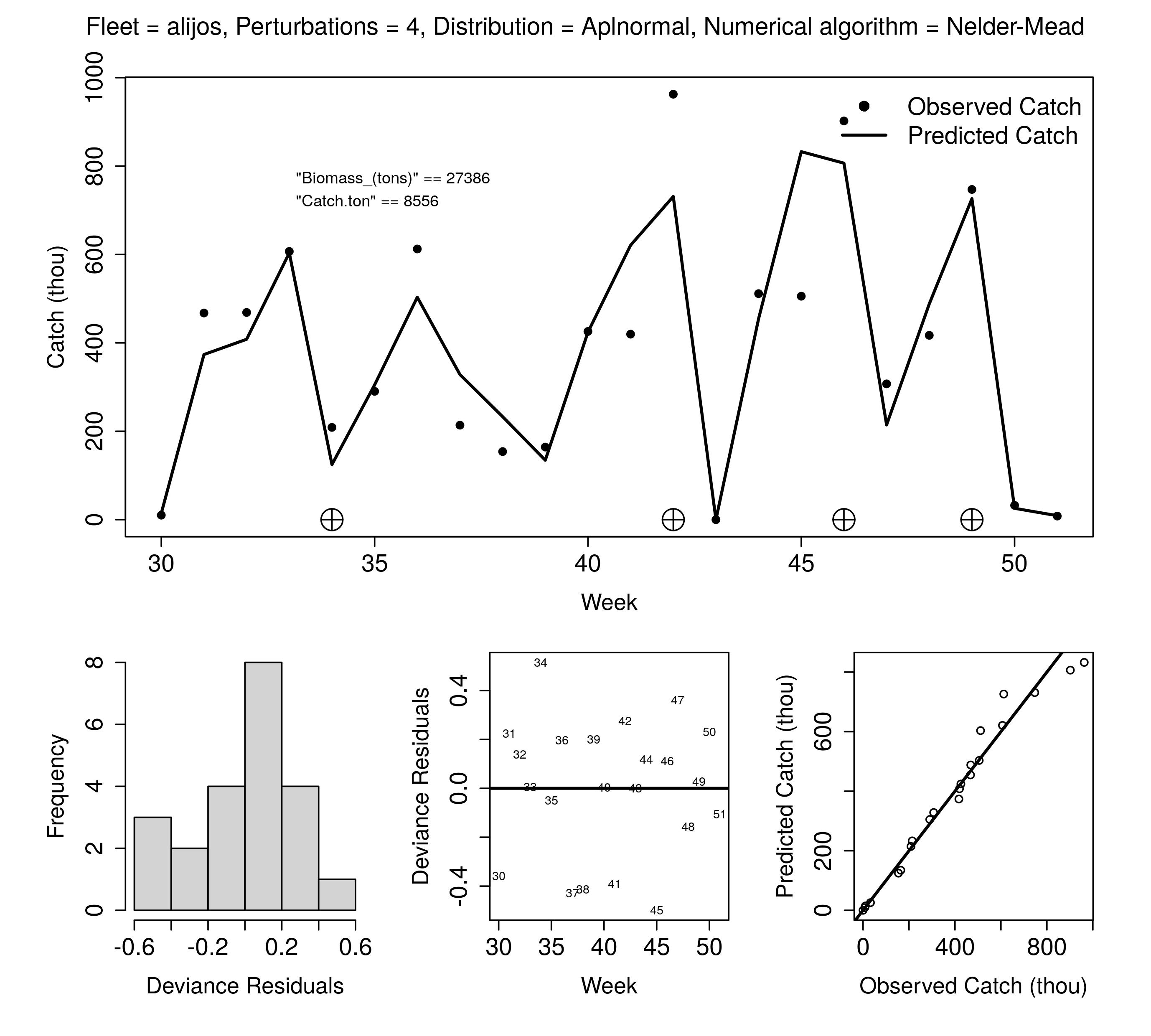

Supplement: S2 File — Model fit to data (top panel; dots: data; line: model) and residual diagnostics (three bottom panels; left: residual histogram; centre: residual cloud; right: quantile-quantile plot) for 22 fishing seasons of O. americanus in Yucatan, Mexico. (ZIP) [file pone.0307836.s002.zip › FigS27CatDynAmer2004.jpg]

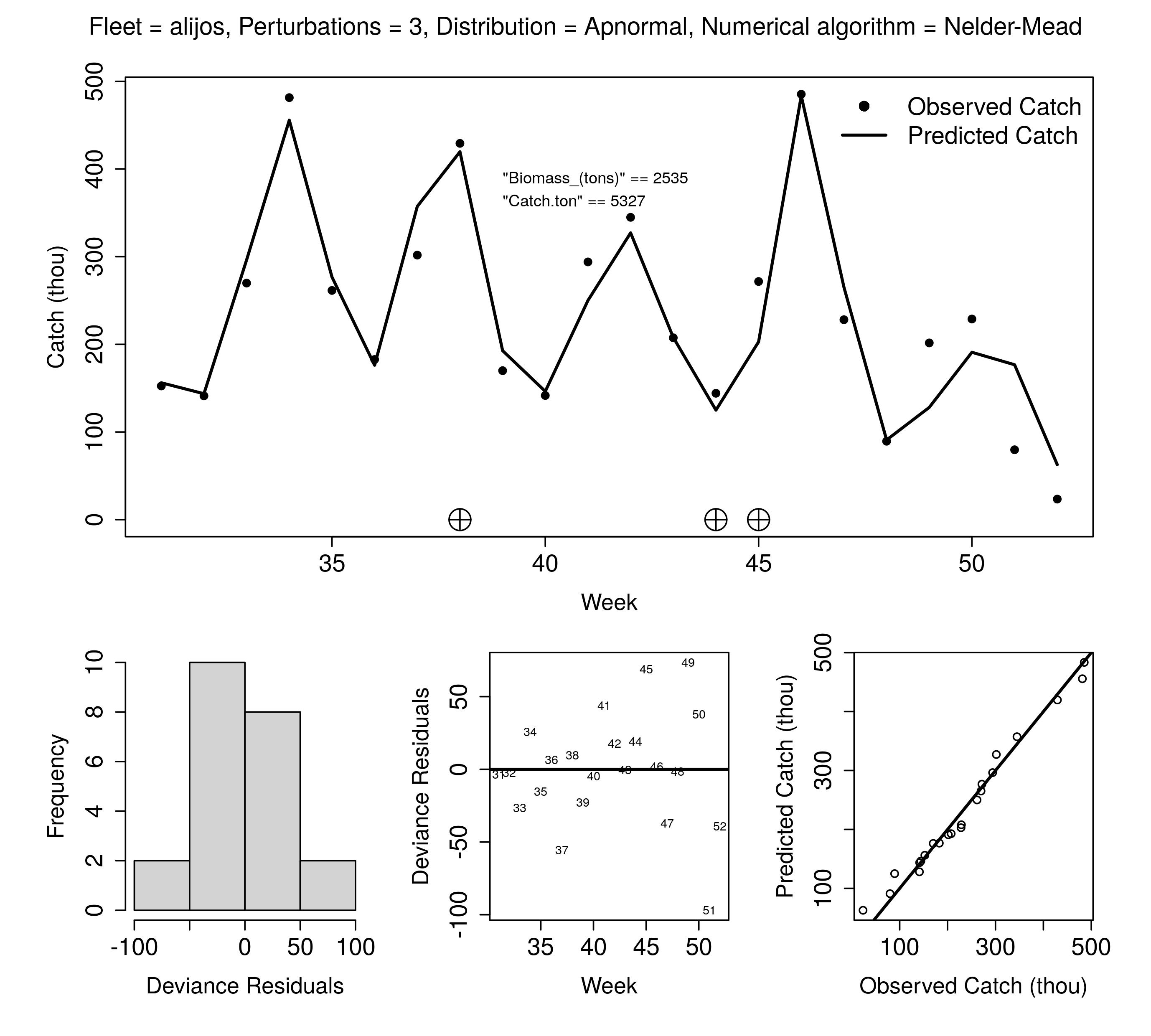

Supplement: S2 File — Model fit to data (top panel; dots: data; line: model) and residual diagnostics (three bottom panels; left: residual histogram; centre: residual cloud; right: quantile-quantile plot) for 22 fishing seasons of O. americanus in Yucatan, Mexico. (ZIP) [file pone.0307836.s002.zip › FigS28CatDynAmer2005.jpg]

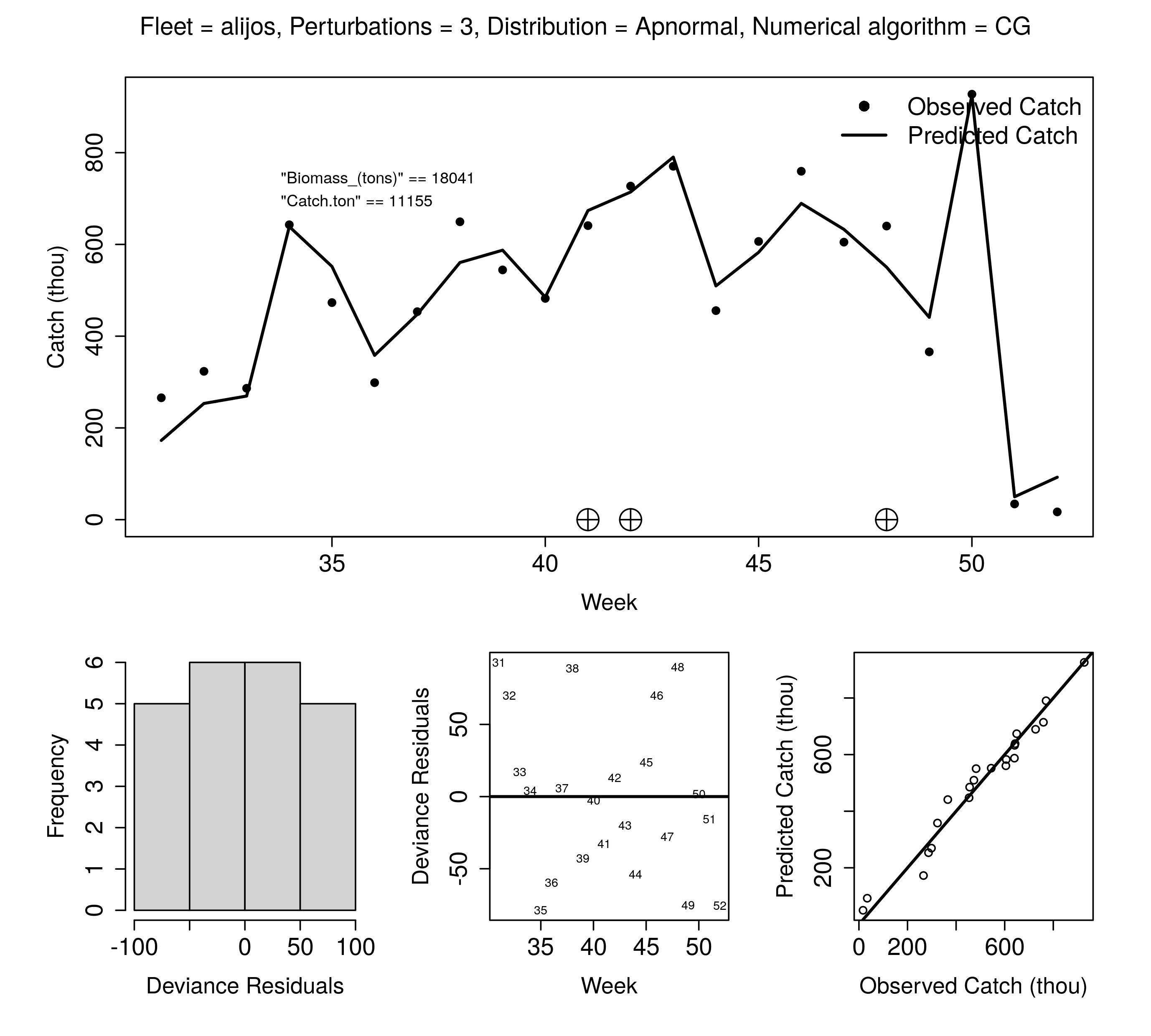

Supplement: S2 File — Model fit to data (top panel; dots: data; line: model) and residual diagnostics (three bottom panels; left: residual histogram; centre: residual cloud; right: quantile-quantile plot) for 22 fishing seasons of O. americanus in Yucatan, Mexico. (ZIP) [file pone.0307836.s002.zip › FigS29CatDynAmer2006.jpg]

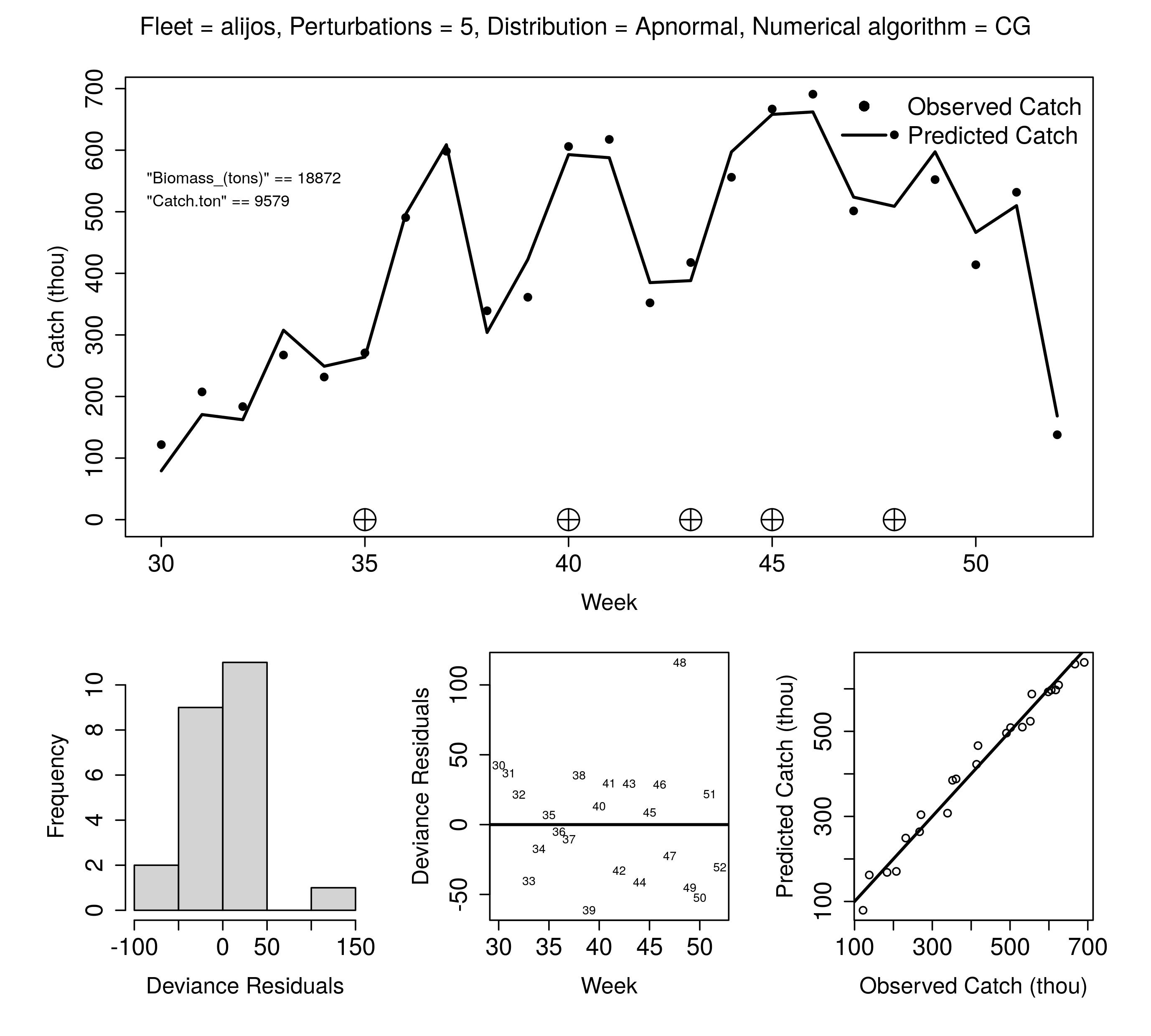

Supplement: S2 File — Model fit to data (top panel; dots: data; line: model) and residual diagnostics (three bottom panels; left: residual histogram; centre: residual cloud; right: quantile-quantile plot) for 22 fishing seasons of O. americanus in Yucatan, Mexico. (ZIP) [file pone.0307836.s002.zip › FigS30CatDynAmer2007.jpg]

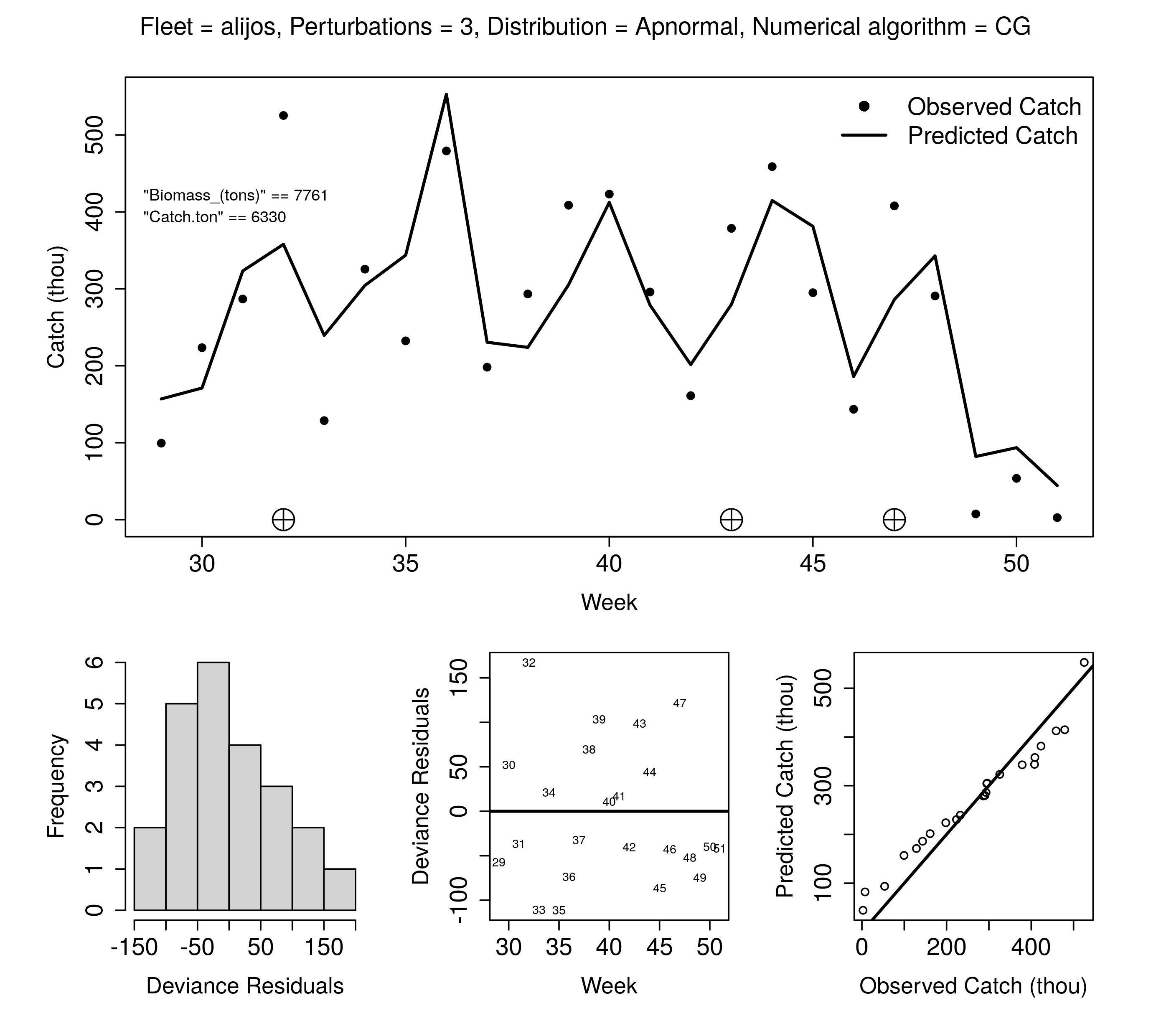

Supplement: S2 File — Model fit to data (top panel; dots: data; line: model) and residual diagnostics (three bottom panels; left: residual histogram; centre: residual cloud; right: quantile-quantile plot) for 22 fishing seasons of O. americanus in Yucatan, Mexico. (ZIP) [file pone.0307836.s002.zip › FigS31CatDynAmer2008.jpg]

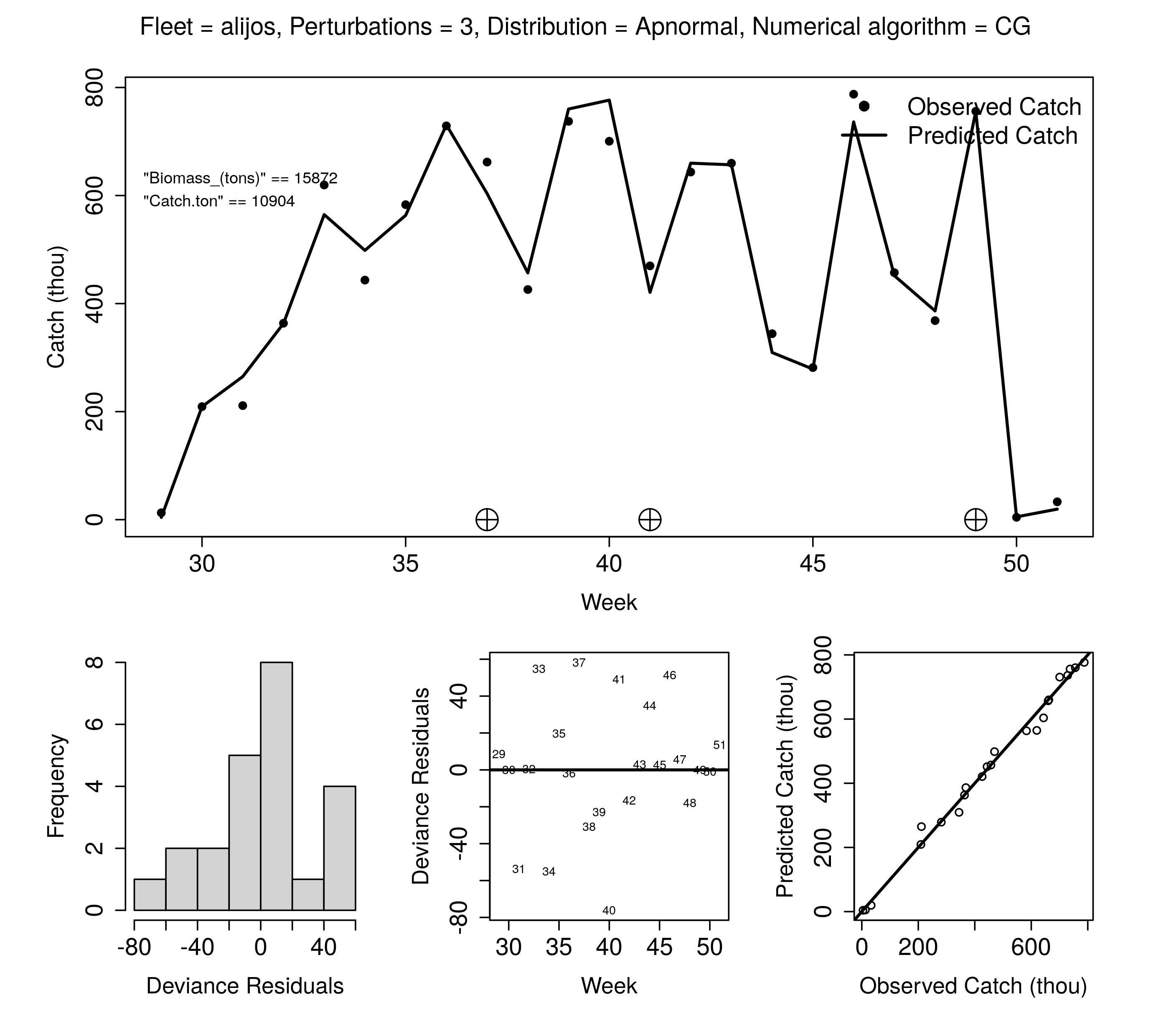

Supplement: S2 File — Model fit to data (top panel; dots: data; line: model) and residual diagnostics (three bottom panels; left: residual histogram; centre: residual cloud; right: quantile-quantile plot) for 22 fishing seasons of O. americanus in Yucatan, Mexico. (ZIP) [file pone.0307836.s002.zip › FigS32CatDynAmer2009.jpg]

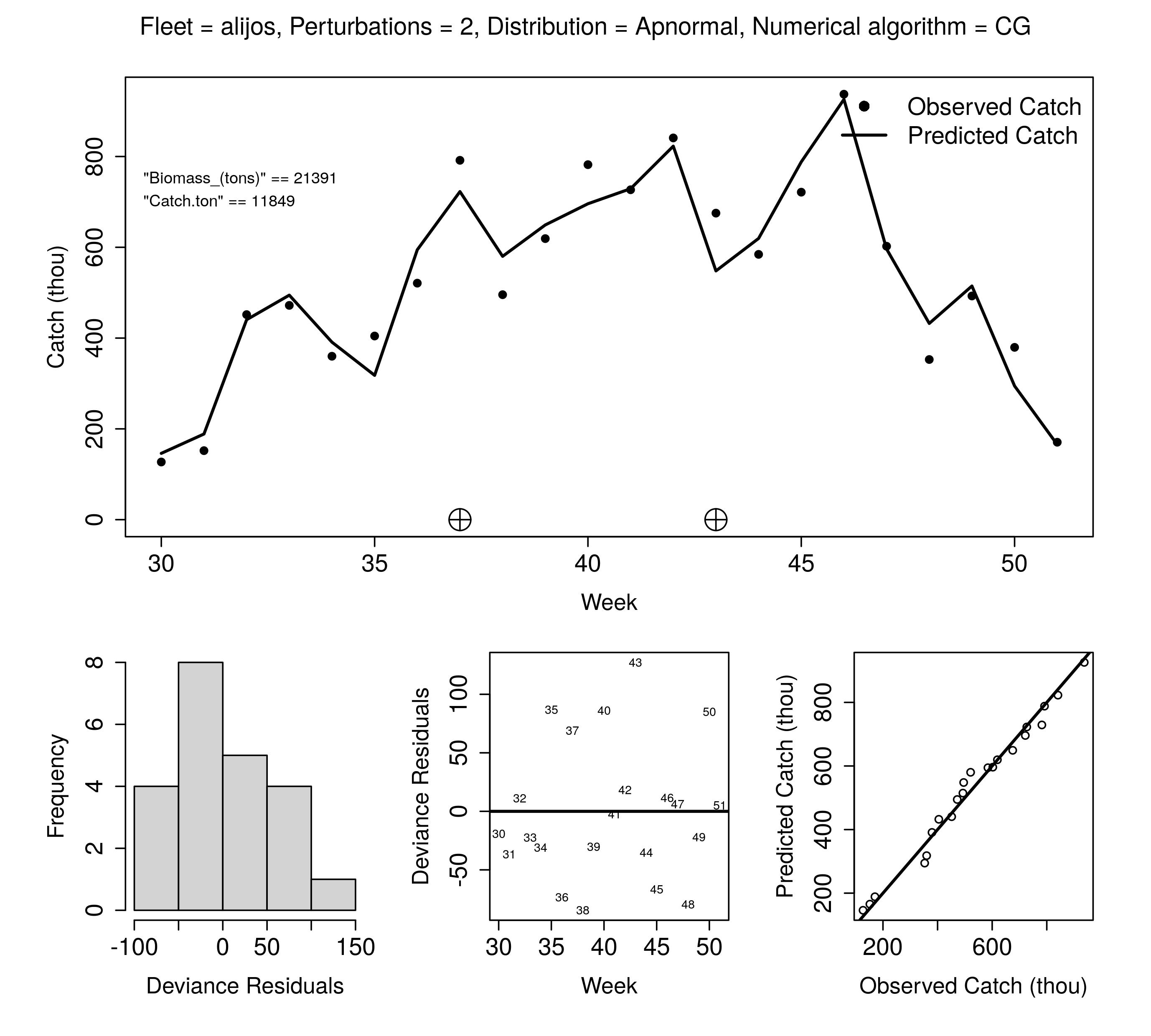

Supplement: S2 File — Model fit to data (top panel; dots: data; line: model) and residual diagnostics (three bottom panels; left: residual histogram; centre: residual cloud; right: quantile-quantile plot) for 22 fishing seasons of O. americanus in Yucatan, Mexico. (ZIP) [file pone.0307836.s002.zip › FigS33CatDynAmer2010.jpg]

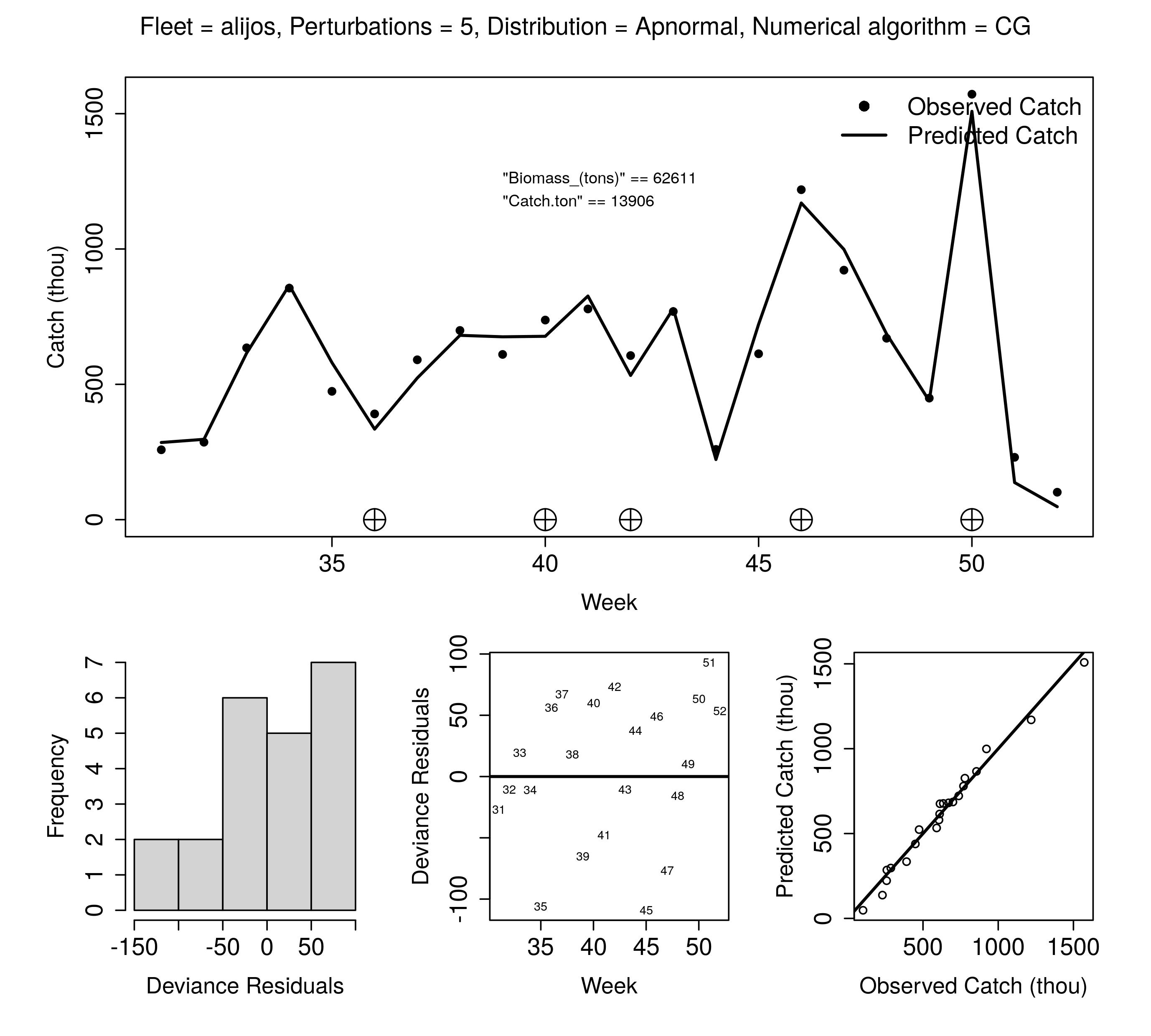

Supplement: S2 File — Model fit to data (top panel; dots: data; line: model) and residual diagnostics (three bottom panels; left: residual histogram; centre: residual cloud; right: quantile-quantile plot) for 22 fishing seasons of O. americanus in Yucatan, Mexico. (ZIP) [file pone.0307836.s002.zip › FigS34CatDynAmer2011.jpg]

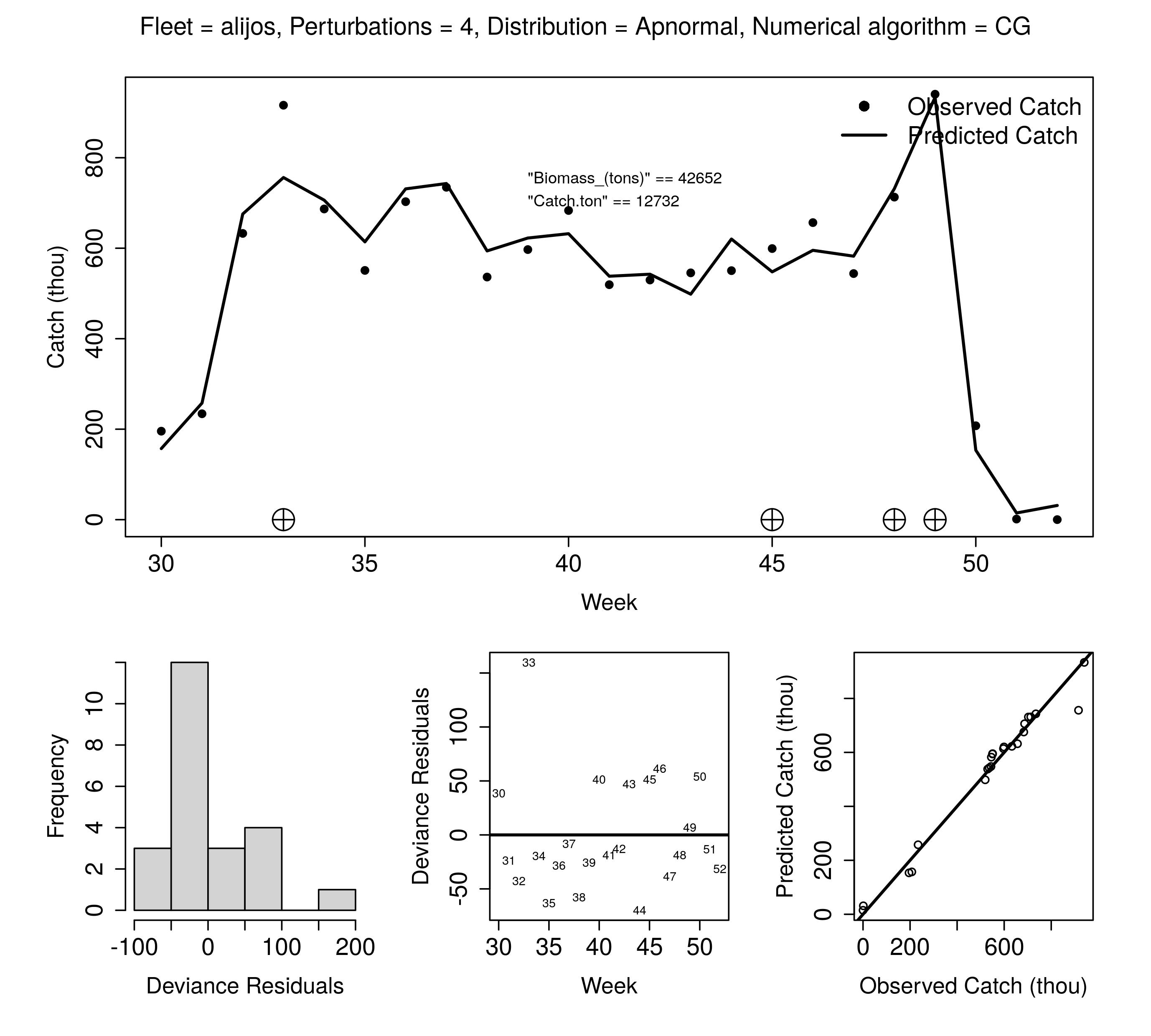

Supplement: S2 File — Model fit to data (top panel; dots: data; line: model) and residual diagnostics (three bottom panels; left: residual histogram; centre: residual cloud; right: quantile-quantile plot) for 22 fishing seasons of O. americanus in Yucatan, Mexico. (ZIP) [file pone.0307836.s002.zip › FigS35CatDynAmer2012.jpg]

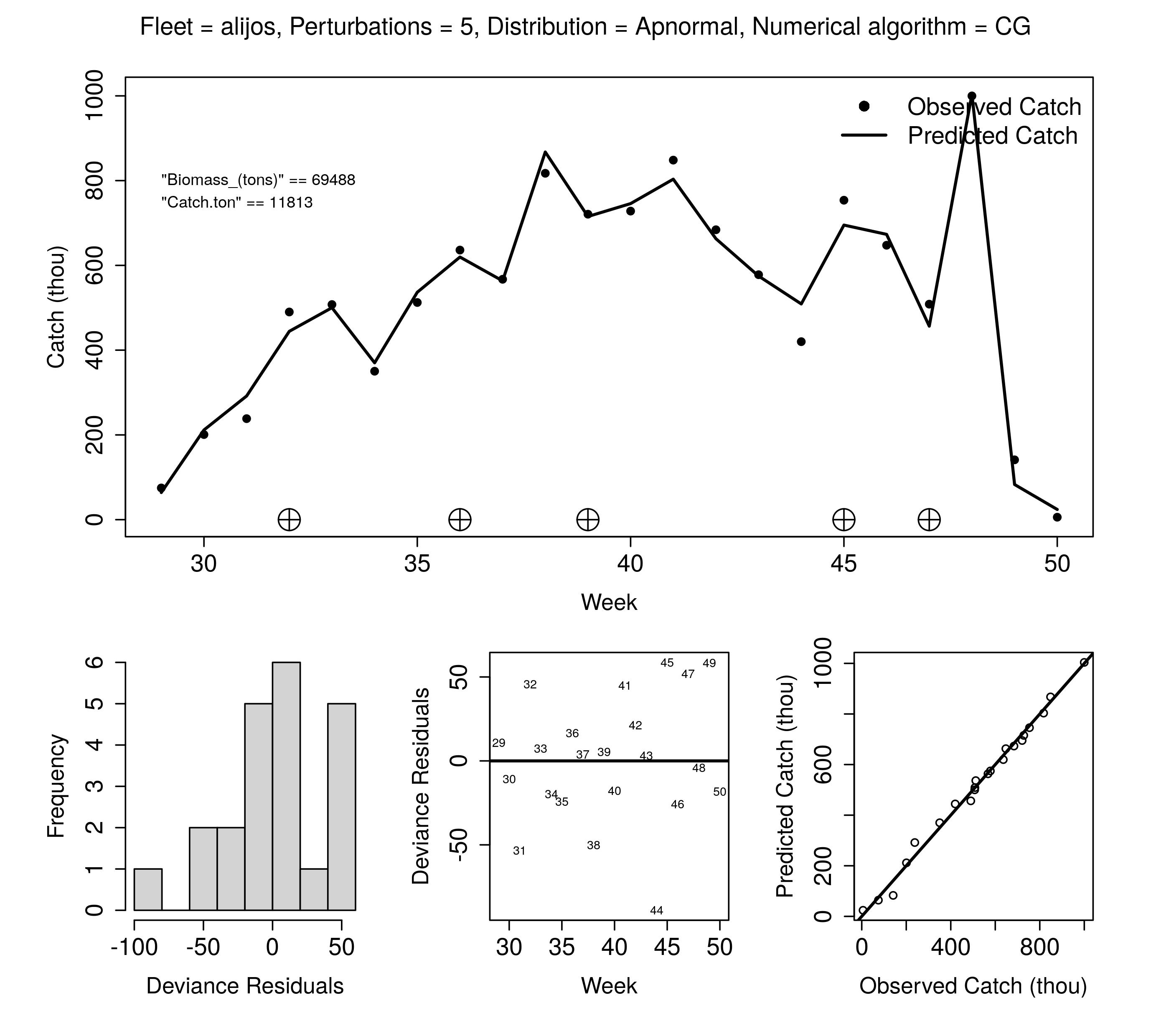

Supplement: S2 File — Model fit to data (top panel; dots: data; line: model) and residual diagnostics (three bottom panels; left: residual histogram; centre: residual cloud; right: quantile-quantile plot) for 22 fishing seasons of O. americanus in Yucatan, Mexico. (ZIP) [file pone.0307836.s002.zip › FigS36CatDynAmer2013.jpg]

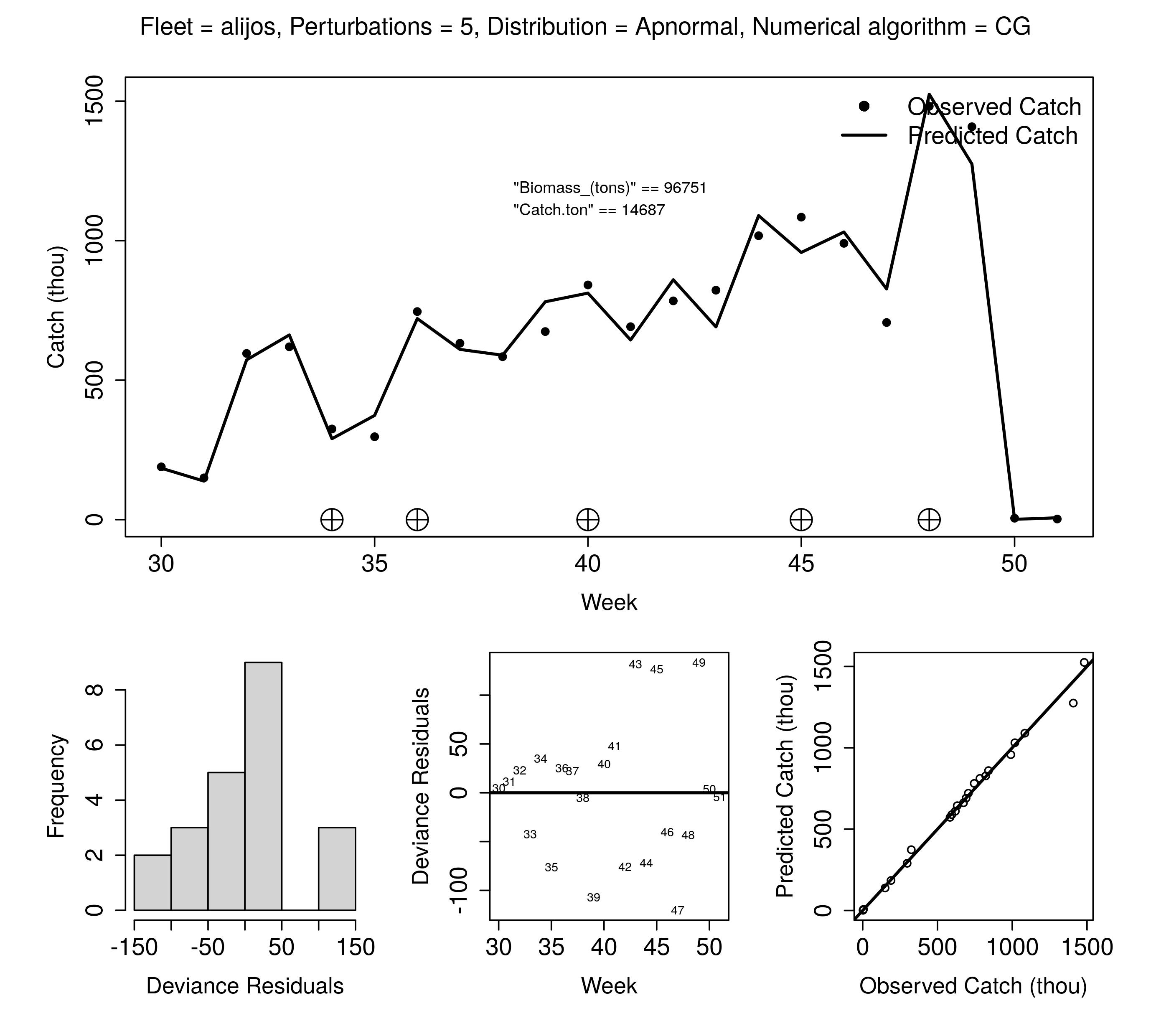

Supplement: S2 File — Model fit to data (top panel; dots: data; line: model) and residual diagnostics (three bottom panels; left: residual histogram; centre: residual cloud; right: quantile-quantile plot) for 22 fishing seasons of O. americanus in Yucatan, Mexico. (ZIP) [file pone.0307836.s002.zip › FigS37CatDynAmer2014.jpg]

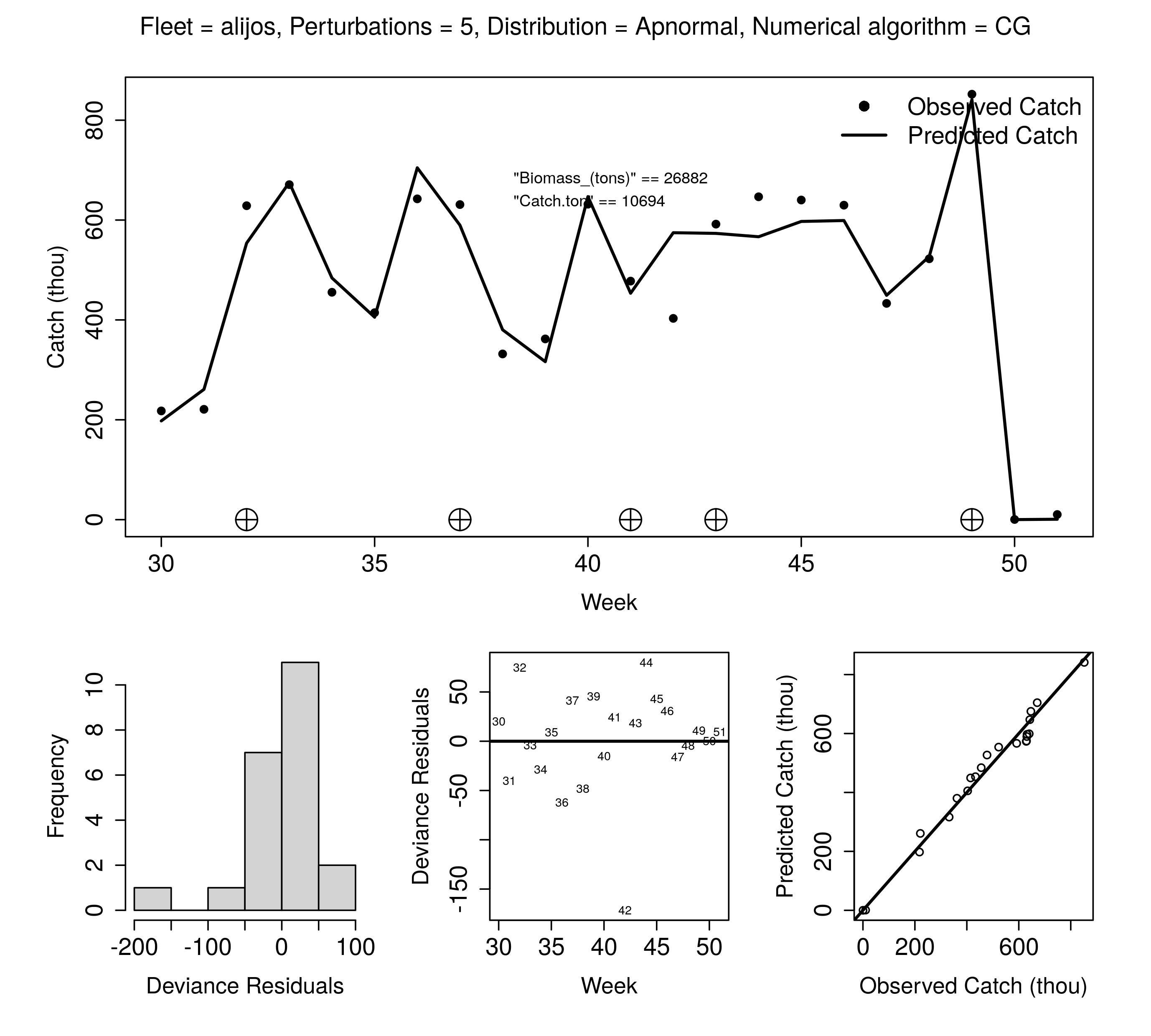

Supplement: S2 File — Model fit to data (top panel; dots: data; line: model) and residual diagnostics (three bottom panels; left: residual histogram; centre: residual cloud; right: quantile-quantile plot) for 22 fishing seasons of O. americanus in Yucatan, Mexico. (ZIP) [file pone.0307836.s002.zip › FigS38CatDynAmer2015.jpg]
