# Supplementary material for: Sustainable fishing harvest rates for fluctuating fish and invertebrate stocks
Source: PLoS One. 2024 Sep 26;19(9):e0307836. doi: 10.1371/journal.pone.0307836 (PMC11426504; doi:10.1371/journal.pone.0307836)
Supplement: S1 Table — Total number of variants, number of converged fits and properties of selected fits, in the fitting of intra-annual generalized depletion models to weekly catch, effort and mean weight of organisms in the octopus fishery of the Yucatan Peninsula, Mexico. R1 to R5 are abundance input pulses. (PDF) [file pone.0307836.s003.pdf]

Table S1.- Total number of variants, number of converged fits and properties of selected fits, in the fitting of intra-annual generalized depletion models to weekly catch, effort and mean weight of organisms in the octopus fishery of the Yucatan Peninsula, Mexico. R1 to R5 are abundance input pulses.

Species	Year	Number of fits	Converged fits	Selected variant name	Selected variant method	Selected variant number	R1 timing	R2 timing	R3 timing	R4 timing	R5 timing
O. maya	2000	20	16	maya2000.P2.1.apln.fit	CG	12	32	43			
O. maya	2001	28	24	maya2001.P3.1.apn.fit	CG	18	35	39	45		
O. maya	2002	24	20	maya2002.P1.1.apn.fit	CG	2	35				
O. maya	2003	20	16	maya2003.P2.1.apn.fit	CG	10	35	41			
O. maya	2004	16	12	maya2004.P2.1.apn.fit	CG	6	38	46			
O. maya	2005	30	28	maya2005.P2.1.n.fit	spg	17	38	38			
O. maya	2006	20	16	maya2006.P1.1.apn.fit	CG	2	41				
O. maya	2007	24	16	maya2007.P3.1.apn.fit	CG	7	34	37	44		
O. maya	2008	20	16	maya2008.P1.1.apn.fit	CG	2	32				
O. maya	2009	24	20	maya2009.P1.2.apn.fit	CG	6	39				
O. maya	2010	16	12	maya2010.P1.1.apn.fit	CG	2	41				
O. maya	2011	32	21	maya2011.P4.1.apn.fit	CG	19	31	38	40	45	
O. maya	2012	36	22	maya2012.P1.1.apn.fit	spg	1	35				
O. maya	2013	16	12	maya2013.P3.1.apn.fit	CG	10	33	40	41		
O. maya	2014	18	10	maya2014.P3.1.apn.fit	spg	7	34	40	42		
O. maya	2015	20	16	maya2015.P2.1.apn.fit	CG	10	35	39			
O. maya	2016	16	6	maya2016.P2.1.apn.fit	CG	3	36	41			
O. maya	2017	18	12	maya2017.P3.1.apn.fit	CG	10	32	49	45		
O. maya	2018	18	14	maya2018.P4.1.apn.fit	spg	12	33	35	37	45	
O. maya	2019	86	78	maya2019.P5.2.apn.fit	CG	68	37	39	41	45	48
O. maya	2020	20	17	maya2020.P3.1.apn.fit	CG	14	35	38	48		
O. maya	2021	44	40	maya2021.P5.1.apn.fit	CG	34	30	32	37	39	42
O. americanus	2000	66	64	amer2000.P5.2.apn.fit	CG	54	36	39	41	47	49
O. americanus	2001	42	36	amer2001.P4.2.apn.fit	CG	22	35	38	42	48	
O. americanus	2002	12	8	amer2002.P1.1.apn.fit	CG	2	40				
O. americanus	2003	50	38	amer2003.P5.1.apn.fit	CG	21	34	37	41	43	51
O. americanus	2004	54	18	amer2004.P4.2.apln.fit	Nelder-Mead	15	34	42	46	49	
O. americanus	2005	27	11	amer2005.P3.2.apn.fit	Nelder-Mead	9	38	44	56		
O. americanus	2006	14	10	amer2006.P3.2.apn.fit	CG	10	41	42	48		
O. americanus	2007	24	21	amer2007.P5.1.apn.fit	CG	20	35	40	43	45	48
O. americanus	2008	8	6	amer2008.P3.1.apn.fit	CG	6	32	43	47		
O. americanus	2009	14	9	amer2009.P3.1.apn.fit	CG	9	37	41	49		
O. americanus	2010	16	12	amer2010.P2.1.apn.fit	CG	6	37	43			
O. americanus	2011	24	20	amer2011.P5.1.apn.fit	CG	18	36	40	42	46	50
O. americanus	2012	20	16	amer2012.P4.1.apn.fit	CG	14	33	45	48	49	
O. americanus	2013	24	19	amer2013.P5.1.apn.fit	CG	17	32	36	39	45	47
O. americanus	2014	60	56	amer2014.P5.3.apn.fit	CG	22	34	36	40	45	48
O. americanus	2015	22	17	amer2015.P5.1.apn.fit	CG	17	32	37	41	43	49
O. americanus	2016	14	10	amer2016.P3.1.apln.fit	spg	9	37	41	42		
O. americanus	2017	48	28	amer2017.P3.3.apn.fit	Nelder-Mead	19	40	43	47		
O. americanus	2018	16	13	amer2018.P3.1.apn.fit	CG	10	38	41	47		
O. americanus	2019	38	32	amer2019.P5.1.apn.fit	CG	30	36	38	41	44	45
O. americanus	2020	12	8	amer2020.P1.1.apn.fit	CG	2	39				
O. americanus	2021	20	16	amer2021.P3.1.apn.fit	CG	14	36	41	45		
Total		1191	912								
