# Supplementary material for: Sustainable fishing harvest rates for fluctuating fish and invertebrate stocks
Source: PLoS One. 2024 Sep 26;19(9):e0307836. doi: 10.1371/journal.pone.0307836 (PMC11426504; doi:10.1371/journal.pone.0307836)
Supplement: Attachment — Submitted filename: PONE-S-24-07756_Rep2Reviewers.pdf [file pone.0307836.s005.pdf]

PONE-D-24-05947
Reply to reviewers (rebuttal letter)

Reviewer #1 – Dr. Chrispine Nyamweya

General comment:
The paper "Sustainable fishing harvest rates for oscillating fish and invertebrate stocks" presents a comprehensive study addressing a critical gap in the management of fish and invertebrate populations subject to fishing. The authors effectively challenge the traditional use of Maximum Sustainable Yield (MSY) as a one-size-fits-all concept for determining harvest rates, by illustrating its inadequacy for populations exhibiting oscillating or unstable dynamics. Instead, they propose the use of Total Latent Productivity (TLP) as a more appropriate measure for setting sustainable harvest rates in such cases. This recommendation is substantiated through a detailed analysis of two octopus species in the Yucatan Peninsula, utilizing a 22-year time series dataset and employing a two-stage stock assessment methodology with time-varying parameters.

Reply: Thanks Dr. Nyamweya for your assessment of our work.

The paper would benefit from a more detailed discussion of the assumptions underlying the two-stage stock assessment methodology and the potential limitations or biases these assumptions might introduce.

Reply: Agreed. We have added a paragraph to the Discussion explaining the three main limitations of the two-stages stock assessment methodology. See L. 791-833.

Overall, the paper, paper is well written and has scientific merit.

Specific comments

The introduction effectively sets the stage for the study, but it could further emphasize the novelty of using Total Latent Productivity (TLP) over Maximum Sustainable Yield (MSY) in the context of fisheries management. Specifically, expanding on how TLP addresses the limitations of MSY in managing oscillating stocks could strengthen the paper's premise. For example, a comparison in lines 50-55 could be deepened with recent case studies illustrating the failure of MSY in similar contexts.

Reply: Agreed. We have added brief extensions to the contrast of MSY and TLP in the Introduction, in the paragraph suggested by the reviewer, and reference to the failure of MSY for small pelagic fish in a recent review paper. See L. 60-64.

line 66: Write “O. vulgaris” on first mention.

Reply: Since this was the first mention of a species by its scientific name, we understood that the reviewer correctly pointed out that we should write the full scientific name. We have done that.

Clarity on the selection criteria for the two-stages stock assessment model, especially the rationale behind choosing specific time-varying parameters, would be helpful. Addressing potential biases these choices might introduce (Lines 230-235) could further solidify the method's validity.

Reply: We have expanded and re-organized the explanation of model selection in subsection **Intra-annual generalized depletion (IAGD) models.** See the 4th paeragraph of this subsection. L. 325-354.

Lines 419-432: While the projections under different harvesting scenarios are insightful, incorporating a discussion on the ecological and economic implications of these projections would enhance the reader's understanding of the practical impacts of adopting TLP-guided management practices.

Reply: Agreed, and thanks for this useful comment. We have expanded paragraph 12th of the Discussion to discuss advantages of the recommended TLP-based limit harvest rates from ecological and economic viewpoints. See L. 764-791.

Lines 580-590: Consider addressing potential challenges in implementing TLP in existing fisheries management frameworks, including the need for capacity building and methodological adjustments, could provide a balanced view of the path forward.

Reply: Agreed, and thanks again, this is another interesting extension of our Discussion. We have added a paragraph at the end of the Discussion (par. 14th) to point out three aspects of implementing TLP-based management in existing fisheries. We have also moved the paragraph about a new Kobe plot to this paragraph because it fits into the topic you raised. See L. 835-878.

PONE-D-24-05947
Reply to reviewers (rebuttal letter)

Reviewer #2 – Anonymous

The manuscript presents an alternative to the MSY concept, average total latent productivity (TLP), as a more appropriate basis for reference points or limits with which to assess and manage fishing of “oscillating” fish stocks. The authors demonstrate the application of average TLP with a stock assessment of two octopus stocks along the coast of the Yucatan peninsula. The methods and results are both comprehensive and rigorously executed and seem appropriate for the authors’ main questions. My main concerns are the prevalence of unclear language and typos throughout the manuscript, and pitfalls of their analysis and results not being addressed (see my General comments as well as detailed comments). No reanalysis is needed, but numerous edits and rewriting parts of the Discussion are needed.

Reply: We apologize for the many typos and lack of clarity in the original ms. but we thank you for your overall positive evaluation. We also want to express our gratitude for a thorough review both from the editorial and conceptual points of views. Thank you very much.

General

Use of “oscillations and unstable”. The use of these terms in the text is confusing. Oscillatory dynamics by definition are stationary (they always revert back to the mean) and regular (fluctuations occur more or less at equal time intervals). As a result, I tend to think of oscillations as stable: however, the authors often group oscillating with unstable dynamics and stocks, and differentiate this group from stable. Clearly defining these terms early on, and double checking their use throughout the manuscript are needed.

Reply: Agreed. We have replaced the terms “oscillatory” and “unstable” with the more general term “fluctuations” in all sections of the ms. where we refer to the general phemomenon of not staying at a single equilibrium point, including the title. We also now define fluctuations as including both oscillations and instabilities in the first sentence of the Abstract, and in the second paragraph of the Introduction.

Sentences that are long and difficult to read and understand. These occur throughout the manuscript and contribute to apparent lack of clarity in some parts.

Reply: We have searched for too long sentences throughout the ms. splitting them to improve clarity.

The Discussion needs text on shortcomings and pitfalls of the methods used in this paper, as well as unaddressed uncertainties. I point out specific results in line specific comments that I see allude to possible shortcomings (e.g. the extreme variability in annual natural mortality estimates, highly precise biomass estimates, changes in fleet characteristics). In general, there are common uncertainties and violated assumptions associated with models that only use fishery-dependent data, which are mostly unacknowledged in the manuscript.

Reply: Agreed partially.

(1) At the request of another reviewer, we added a paragraph to the Discussion explaining three limitations of our methodology. See L. 792-834.

(2) However, we do not consider that highly precise biomass estimates are artificial or a result of violations of assumptions. Biomass estimates from depletion models for *O. americanus* had a mean CV of 53%, maximum CV of 272% and minimum CV of 8% across the 22 years of estimates, and biomass estimates from *O. maya* had a mean CV of 23%, maximum CV of 130% and minimum CV of 6% across the 22 years of estimates. Also Fig. 4 shows reasonable error bands for the biomass time series estimated by the Pella-Tomlinson model. One advantage of our methodology is that it has a self-weighting likelihood function, which by virtue of using the standard error of estimation of each biomass estimates, up-weights precise biomass estimates and down-weights imprecise biomass estimates when fitting Pella-Tomlinson's model to biomass and annual landings data. This feature of the likelihood in eq. 7 permits more precise estimates of the biomass time series from the Pella-Tomlinson model.

Detailed comments

Lines 8. Do you mean limit reference points? Or limiting stock biomass? This wording (e.g. "limit stock biomass") is unclear to me. Please rephrase.

Reply: Re-phrased. See L. 7-10.

Lines 8-11: With regards to conventional manuscript structure and style, stating the purpose of the study usually comes at the end of the Introduction. Additionally, this sentence does not flow well from the preceding sentences, which outline the widespread use and importance of MSY. I expected the last sentence to introduce well-known challenges and pitfalls that counter the use of MSY (but instead says "TLP should be used instead" without any reasoning yet)

Reply: OK. Some authors want to go straight to the point at the start of the Introduction but our attemtp at doing that did not yield good results. So we deleted this sentence. But we do not want to start pointing out to all other criticisms of the MSY because we feel that those are largely resolved by using the MSY as a generator of limit reference point, instead of a desired harvest rate.

13. Here and elsewhere where "instability" is used, as well as "oscillations". I understand these concepts from a mathematical perspective, but I expect your audience will be mostly non-mathematicians. Or perhaps I am misinterpreting these terms myself. Either way, I suggest these terms be clearly and concisely defined (here or elsewhere in the Introduction). The next sentence somewhat defines instabilities (starting line 14), but should be rephrased more directly for clarity (e.g. "Instabilities are ...."). Same for oscillations ("Oscillations are....").

Reply: Agreed. We have now more precise definitions and have introduced the term "fluctuations" to refer to both, oscillations and irregular instabilities. See L. 11-19.

26. I think this should be "Oscillating or unstable…"

Reply: Agreed, but now we use fluctuations to encompass both, oscillations and irregular instabilities. See L. 28.

36. Using "instabilities" alone is unclear. Please be more specific (e.g. do you mean irregular population fluctuations? Or something else?).

Reply: Clarified. See L. 38.

41-42. I am unsure what "behavioural dynamics … is stable" means.

Reply: In the quoted paper (Halloway et al., 2020) authors use the term ‘behavioral dynamics’ to refer to the change in time of the degree of cooperation inside a group, as opposed to population dynamics, that describe the change in time of population size. They find both dynamics are disconnected to argue that obligate cooperative systems should be unstable at the population dynamics level. This is outside the scope of our paper and not really necessary so we feel that it is better to take out the reference.

46. Unsure what “viable oscillating or unstable stocks” are. Please clarify.

Reply: We have re-phrased that part to improve clarity. What we are saying is that fluctuating stocks (either oscillatory or irregularly unstable) have positive growth rate when they are increasing from low biomass to high biomass, and they have negative growth rates when they are decreasing from high biomass to low biomass. In the mid- to long-term positive growth rates have to be higher on average than the absoute value of negative growth rates, because if the opposite happens, stock biomass will collapse to zero. So ‘viable’ means that in the mid- to long-term positive growth rates are larger than (absolute value) negative growth rate so that biomass remains positive. We explained this is a short format in the revised ms. See L. 43-48.

In other words:
In stocks with stable dynamics, what we want is that the growth rate is always positive.
In fluctuating stocks the equivalent goal is that the mid- to long-tern average growth rate (averaging over positive and negative growth rates) is positive.

55-56. This sentence is unclear. I think there are extra words that don’t make sense, or missing words.

Reply: Re-phrased to clarify. See L. 54-56.

56. Typo in “iscillating”.

Reply: Replaced with ‘fluctuating’, see L. 56.

67. Remove “found” (revised sentence is then: “…that have been connected to…”

Reply: Done, see L. 71.

77-81. This sentence is difficult to understand. Please rephrase for clarity.

Reply: Done, see L. 81-86.

81-85. I do not see how this tests your hypothesis, although this may be because I do not fully understand what is “unstable dynamics”. I think clearly defining unstable (and differentiating from stable and oscillating) will address this confusion and other comments I have (see comment for Line 13).

Reply: See the changed text at the end of Introduction, L. 86-88.

90. Change “originated” to “originating”.

Reply: Done, L. 93.

96. Can simplify “recurrent incidence” to “recurrence”.

Reply: Done, L. 99.

100. I am unsure what is a “target resource.” Please clarify.

Reply: We meant non-bycatch, but it was better to just delete that fragment, see L. 103.

103. “new technological capabilities” is vague. Recommend being more specific (e.g. is it in the fishing gear? The boats?).

Reply: Agreed, see L. 105-108.

105. “…total production…” of what exactly?

Reply: See L. 108.

116-119. This sentence is confusing and may be improved by splitting into two sentences. Either way, please revise for clarity.

Reply: Done, see L. 120-123.

124. “Those fleets….”: Do you just mean the 2nd fleet? Or both fleets? Please revise for clarity.

Reply: Done, see L. 127-129.

133. You can split this sentence at “… in similar abundances [8,9]. Hence, …”. This would also improve the clarity of this sentence.

Reply: Done, see L. 138-139.

138. Remove the “,” between “length” and “or”.

Reply: Done, see L. 143.

148. I do not think this “,” is necessary here.

Reply: Done, see L. 153.

156. “…adding over…” can be simplified to “…of…”.

Reply: Done, see L. 161.

158. Unsure what “…on the multiplicative effect of carrying a number of alijos that conduct the fishing” means. Please revise for clarity.

Reply: Rephrased for clarity, see L. 162-164.

159. Please change “contained” to “contains”. And elsewhere, check for consistency in your use of verbs, specifically whether they are past or present. Throughout the methods, usage has switched between past and present tense, which I think has also contributed to some of the lack of clarity noted so far.

Reply: Done, and throughout the text for M & M we’ve checked for consistency in verb use, keeping/switching to past tense when we describe things we did and to present tense when we described things that still are. See L. 164.

173. “For stock assessment purposes, …” is unclear and leaves the reader guessing as to what purpose (and if there is more than one). I understand the implication (a discrete-time model needs equal time steps), but I assume other readers may not and thus recommend being more specific here.

Reply: Agreed, clarifications added, see L. 178-179.

180. I do not know what is “… an error of time attribution…”. Did you throw out the trips >1 week? Or include them any ways? Please rephrase this sentence.

Reply: All fishing trips were included. It’s just that some trips that lasted more than one week were assumed to happen entirely in the 1st week in order to have a weekly time step for the depletion models. Another option was to group the data at monthly time steps but that decision would have precluded estimation of time-varying parameters in the depletion model and a great loss of degrees of freedom to estimate parameters (due to having many more weeks than months in a fishing season). So we judged that the loss due to erroneous time attribution of some fishing trips was minor compared to the loss of aggregating the data at the monthly time step. See clarifications in L. 186-199.

183-186. This sentence is somewhat confusing as well. I think there are too many ideas trying to be presented at once that don’t necessarily make sense when combined together (e.g. “weekly time step stock assessment employed”, and “allows direct estimation of annually-varying fishery”). Recommend revising.

Reply: The clarified text between L. 186-199 has resolved this excess.

196. Omitting “…from a model for the progression…” would streamline this sentence while still allowing it to say what it currently says (but a little more clearly).

Reply: Agreed. Done. See L. 209.

205. Are you resampling directly from the observations (or errors from the loess fit), OR are you generating new random values from the truncated normal based on the mean and standard deviation from your fit?

Reply: The second clause after OR. See clarifications between L. 216-221.

219-224. This is another very long sentence that is difficult to read. More generally, I suggest to carefully reread the entire manuscript, identify sentences where 3 or more commas are used and nothing is being listed or presented as steps in a process (e.g. lines 214-216 is listing different time series, and this is fine), and break these sentences down further into multiple sentences.

Reply: Agreed. The sentence has been split and the last part (“coming across as excellent …”) has been deleted. See L. 232-236.

244. For the variance-covariance matrix, is a symmetric, uncorrelated structure used? It seems this would be the only option if you are only using the estimates of the standard error. Please clarify either way.

Reply: Yes, the variance-covariance matrix of the vector of biomass MLEs is symmetric and diagonal by design, because each biomass estimate come from separate fitting of depletion models to each fishing season. So the correlation between biomass estimates of different years (seasons) is zero. This has been clarified. See L. 257-260.

261. “Therefore” is unnecessary here.

Reply: Disagree. The fact that the season does not include the spawning period has the consequence that spawning emigrations cannot be estimated. It is a consequence, so “therefore”.

Eqn 1. Is “Rj” just representing recruitment? If there is no other “abundance inputs”, I would just refer to this term as “recruitment” here and throughout the manuscript. This would make clear to the reader there are no other “abundance inputs” considered (e.g. immigrants). Also, perhaps aggregate the bounds on the terms below the equation to make for easier reading (e.g. “t, k, alpha, beta, M, and Rj > 0”)

Reply: Disagree on the first part and agree on the second part. We cannot call all those inputs “recruitment” to simplify exposition. There are abundance inputs that are not recruitment of octopus growing to the size retained by fishers. These other abundance inputs are displacements of part of the fleets to fishing ground previously not fished, and these movements by the fleet are equivalent to immigrants to the vulnerable part of the stock. This is what happens when modelling the catch at rapid time steps as it is done with depletion models: things like part of the fleet moving to un-used fishing grounds become important to the modelling. These inputs of abundance are all included in the back-calculation of biomass at start of season in eq. 2. The second part of the suggestion, to group term that have similar bounds to ease reading, was adopted, see eq. 1.

282-290. Are there objective criteria for identifying the “most positive residuals” that are “standing-out”?

Reply: If you are asking about objective criteria such as number of standard erros above zero, the answer is no. But we ID the weeks of the most positive residuals as the ones that are highest in a residual cloud plot. Usually they stand out egregiously but sometimes these residuals are fairly close to the clould of residuals. An objective test of the reality of those standing out residuals is applied post hoc. We fit models with and without those residuals as abundance inputs and then select the best variants using tools like the AIC.

334. I believe “determined” should be in the present-tense (determine).

Reply: Yes, corrected. See L. 356.

400. Typo (cominf grom).

Reply: Corrected, L. 422.

438. I suggest having a short sentence before this one clarifying that 44 variants were selected from the 912 converged models based on the model selection criteria (if this is indeed true).

Reply: Yes, short sentence added as suggested. See L. 460-461.

454-456. This is extraordinary variation in natural mortality for both species; for example, a high M of 6.4 1/yr for O. maya. implies a survival proportion of <0.01. There should be some Discussion text devoted to the biological realism of these parameters (and others), and what this implies about the underlying assumptions of the IAGD model and the resulting absolute biomass estimates.

Reply: Agreed. We have added a paragraph in the Discussion (10th par.) discussing these results. Essentially we think that strong density-dependence in the spawning stock and recruitment relationship causes large variations in M. Years of high M are caused by strong cannibalism. We provide two pieces of evidence for this hypothesis. See L. 733-755.

461. This precision should be considered in the context of the underlying assumptions of the IAGD. This is my first encounter with this specific methodology and I suspect there are some fairly strong assumptions about how catches represent both population levels and population dynamics.

Reply: There are now close to 20 papers published with this methodology, in journals such as the ICES JMS, Fisheries Research, Ecological Modelling, Frontiers in Marine Science. Fishery Bulletin, Transactions of the American Fisheries Society, Marine Policy, Fisheries Management and Ecology, and Scientia Marina. The methodology has been applied to all kinds of fisheries, small and large scale, artisanal and industrial, coastal and far seas, for fish and invertebrates, in papers and technical reports. Assumptions and limitations of generalized depletion models have been thoroughly tested and discussed. In addition, the Australian Stock Assessment toolbox (https://toolbox.frdc.com.au/toolbox/) lists the software used in this work (R package CatDyn, created and mantained by one of us, RR-U) in the top category: “Supported and recommended”.

Apart from those facts, consider the 44 plots that we put in Supplementary Material, showing the fit of the model to the data. They show generally excellent fits. The model follows the data closely in nearly all seasons.

The ultimate reasons for the success of this type of approach to modelling fisheries data and to routinely provide high precision *M* estimates is that it uses high time-resolution data, weekly data in our case, which brings abundant degrees of freedom and ups and downs in catch (contrast) to estimate parameters, even parameters that are difficult to estimate in data-rich, annual time step stock assessment models, such as *M*.

Nevertheless, *M* is the most difficult parameter to estimate in this kind of models, as in those running at slow time steps. A lot of the discarded variants (we fitted 1,191 variants in total) have non-sensical estimates of *M*.

At the request of another reviewer, we added a paragraph with limitations and assumptions of the methodology, see the 13th par. of Discussion, L. 792-834.

498. Suggest omitting “variant” in front of “surplus production models..”. The word “variant” is singular, while multiple “models” are referred to.

Reply: Done, see L. 522. We searched for other cases of this mismatch in the ms. and did not found any other.

530-531. This is a consequence of the information going in (biomass and SE estimates from the IAGD models, which have a lot of observations being fit). I am generally skeptical of such precision because these low values are not seen in other data-rich stock assessments. Some part of the Discussion should address this, if such text does not already exist.

Reply: We are also generally skeptical of CVs less than 10% in any stock assessment. This methodology yields precise estimates with long time series of landings and biomass estimates while short time series yield the opposite, imprecise estimates. See the new paragraph (13th) added at the request of another reviewer, with references to other published applications of this methodology.

The other crucial factor in having precise estimates for the Pella-Tomlinson model with this methodology is the marginal-estimated likelihood, which automatically up-weights precise estimates of biomass and down-weights imprecise ones.

This is not usually done in conventional fits of the Pella Tomlinson model to CPUE indices of abundance, where all year coefficients for relative abundance are treated as equal.

579. Typo here: "… relevance of this argumentmanner"

Reply: Corrected. See L. 601-602.

579. Unsure what is "W" as I do not recall it being previously defined.

Reply: Another corrected typo. See L. 602-603.

581. Use of "the" in "is the average TLP the valid estimate." As written, this proposes TLP as a definitive solution (which may or may not be what the authors intend). I suggest replacing with "… TLP a valid estimate", as there exist alternative reference point estimators (MEY, some proportion of virgin biomass, stochastic MSY from Bordet and Rivest 2014, etc.). Bordet, C. and Rivest, L.-P. (2014) A stochastic Pella Tomlinson model and its maximum sustainable yield. Journal of Theoretical Biology 360, 46–53

Reply: Agreed. We've changed the statement as suggested. See L. 603-606. And of course, it was not our intention to present the TLP as **the** solution. We are aware of Bordet and Rivest paper and it is quoted in our ms., although for reasons other than alternatives to the classic MSY.

606-636. While it is indeed well established many fish stocks exhibit "unstable" dynamics (whether this implies nonstationary productivity, highly variable recruitment, etc.), I think the conclusion that "…the average TLP should replace the MSY…" is not convincing without some discussion on other proposed or used alternative reference point estimators (as noted in my comment for line 581, among others not mentioned). Additionally, management procedures for most fish stocks use harvest control rules and other tools for computing fishing pressure targets or limits, which MSY reference points may or may not be used to configure. For example, Fmsy may be used to set a target fishing pressure above a pre-specified biomass threshold in a harvest rule. Ideally, I'd rather see this paragraph streamlined/shortened, and another paragraph added discussing average TLP compared/contrasted with other common reference points.

Reply: The Discussion is already long, because of added and expanded paragraphs, so comparing the TLP to other reference points such as escapement biomass, proportion of un-fished spawning biomass, and other reference points not coming from the MSY, seems like a new research project.

However, you are correct in that our conclusion was too strong as it was worded. So in this paragraph (5th par. of Discussion) we are inserting disclaimers to delimit our assertion to the subset of reference points derived from the MSY. See L. 629-631, and L. 662.

638: The Kobe plot was created much earlier than 2017. Double check references.

Reply: We couldn't find any reference to the original presentation of the phase diagram used in fisheries management. So we re-phrased that part to say that the diagram was just named after the meeting in Kobe, not that it was first presented there. See L. 663.

654-658. Another long and difficult to read sentence.

Reply: The sentence was split. See L. 680-684.

667-682: In general, changes in fishing fleet characteristics (whether technology creep, trends in fishing behavior, etc.) undermine biomass estimates in these type models, where only catches and effort are the only sources of information. I was glad to see that fleet changes over time are acknowledged. But, as you imply in the last sentence that changes in fishing fleet coverage "may underlie fluctuations in total biomass", what does this imply about the validity of the TLP estimates? If biomass is actually a signal of changes in fishing coverage, then the Pella-Tomlinson model parameters will be biased. At the very least, there needs to be more discussion of assumptions and likely violations of those assumptions in both models (and what this implies about the estimates).

Reply: Yes. This is an issue that concerns just one species (*O. americanus*) and that was extensively discussed inside our group. We agree with you that it may affect accuracy of parameter estimates during the pre-expansion period. We have added a brief discussion of the impact of fleet changes at the end of the paragraph. See L. 708-715.

722: Is this "Conclusion" subtitle misplaced?

Reply: This was a leftover from the Latex template. Deleted.

Fig. 2. Please add subplot labels (e.g. a), b), c), etc.) and increase the text size within the plots (the labels for the y-axis). Also, the estimates of the k parameters (catchability) from the IAGD models should be included.

Reply: Done. See the new Fig. 2.

Fig. 3. This plot is difficult to read, and I am unsure what the main takeaway is. A suggested improvement is to remove the x-axis ticks and labels of all the inner plots (and only display them at the bottom). An alternative and better suggestion is to just have one plot for each species (side-by-side) with all years in each plot. Then the lines can be shaded or colored along a gradient representing the progression from 2002-2021. It will be easy to see trends and shifts over time then (e.g. lines become lower and lower as time progresses).

Reply: Done. See the new Fig. 3.
We used the following:
colfunc <- colorRampPalette(c("red","yellow","springgreen","royalblue"))
and then for each year we defined the line color as
col=colfunc(22)[x],
where x goes from 1 (2000) to 22 (2021)

Fig. 4. I think it is worthwhile to show the MSY value(s) for reference.

Reply: Agreed. Done. See the new Fig. 4
