# Supplementary material for: Sustainable fishing harvest rates for fluctuating fish and invertebrate stocks
Source: PLoS One. 2024 Sep 26;19(9):e0307836. doi: 10.1371/journal.pone.0307836 (PMC11426504; doi:10.1371/journal.pone.0307836)
Supplement: Attachment — Submitted filename: Review comments.docx [file pone.0307836.s004.docx]

General comment:

The paper "Sustainable fishing harvest rates for oscillating fish and invertebrate stocks" presents a comprehensive study addressing a critical gap in the management of fish and invertebrate populations subject to fishing. The authors effectively challenge the traditional use of Maximum Sustainable Yield (MSY) as a one-size-fits-all concept for determining harvest rates, by illustrating its inadequacy for populations exhibiting oscillating or unstable dynamics. Instead, they propose the use of Total Latent Productivity (TLP) as a more appropriate measure for setting sustainable harvest rates in such cases. This recommendation is substantiated through a detailed analysis of two octopus species in the Yucatan Peninsula, utilizing a 22-year time series dataset and employing a two-stage stock assessment methodology with time-varying parameters.

The paper would benefit from a more detailed discussion of the assumptions underlying the two-stage stock assessment methodology and the potential limitations or biases these assumptions might introduce.

Overall, the paper, paper is well written and has scientific merit.

Specific comments

The introduction effectively sets the stage for the study, but it could further emphasize the novelty of using Total Latent Productivity (TLP) over Maximum Sustainable Yield (MSY) in the context of fisheries management. Specifically, expanding on how TLP addresses the limitations of MSY in managing oscillating stocks could strengthen the paper's premise. For example, a comparison in lines 50-55 could be deepened with recent case studies illustrating the failure of MSY in similar contexts.

line 66: Write “O. vulgaris” on first mention.

Clarity on the selection criteria for the two-stages stock assessment model, especially the rationale behind choosing specific time-varying parameters, would be helpful. Addressing potential biases these choices might introduce (Lines 230-235) could further solidify the method's validity.

Lines 419-432: While the projections under different harvesting scenarios are insightful, incorporating a discussion on the ecological and economic implications of these projections would enhance the reader's understanding of the practical impacts of adopting TLP-guided management practices.

Lines 580-590: Consider addressing potential challenges in implementing TLP in existing fisheries management frameworks, including the need for capacity building and methodological adjustments, could provide a balanced view of the path forward.
